# Impact of the COVID-19 pandemic on antipsychotic prescribing in individuals with autism, dementia, learning disability, serious mental illness or living in a care home: a federated analysis of 59 million patients’ primary care records in situ using OpenSAFELY

**DOI:** 10.1136/bmjment-2023-300775

**Published:** 2023-09-15

**Authors:** Orla Macdonald, Amelia Green, Alex Walker, Helen Curtis, Richard Croker, Andrew Brown, Ben Butler-Cole, Colm Andrews, Jon Massey, Peter Inglesby, Caroline Morton, Louis Fisher, Jessica Morley, Amir Mehrkar, Sebastian Bacon, Simon Davy, David Evans, Iain Dillingham, Tom Ward, William Hulme, Chris Bates, Jonathan Cockburn, John Parry, Frank Hester, Sam Harper, Shaun O'Hanlon, Alex Eavis, Richard Jarvis, Dima Avramov, Nasreen Parkes, Ian Wood, Ben Goldacre, Brian Mackenna

**Affiliations:** 1 Pharmacy, Oxford Health NHS Foundation Trust, Oxford, UK; 2 Nuffield Department of Primary Care, Oxford University, Oxford, UK; 3 TPP-UK, Leeds, UK; 4 EMIS Group PLC, Leeds, UK

**Keywords:** COVID-19, delirium & cognitive disorders, adult psychiatry, impulse control disorders

## Abstract

**Background:**

The COVID-19 pandemic affected how care was delivered to vulnerable patients, such as those with dementia or learning disability.

**Objective:**

To explore whether this affected antipsychotic prescribing in at-risk populations.

**Methods:**

With the approval of NHS England, we completed a retrospective cohort study, using the OpenSAFELY platform to explore primary care data of 59 million patients. We identified patients in five at-risk groups: autism, dementia, learning disability, serious mental illness and care home residents. We calculated the monthly prevalence of antipsychotic prescribing in these groups, as well as the incidence of new prescriptions in each month.

**Findings:**

The average monthly rate of antipsychotic prescribing increased in dementia from 82.75 patients prescribed an antipsychotic per 1000 patients (95% CI 82.30 to 83.19) in January–March 2019 to 90.1 (95% CI 89.68 to 90.60) in October–December 2021 and from 154.61 (95% CI 153.79 to 155.43) to 166.95 (95% CI 166.23 to 167.67) in care homes. There were notable spikes in the rate of new prescriptions issued to patients with dementia and in care homes. In learning disability and autism groups, the rate of prescribing per 1000 decreased from 122.97 (95% CI 122.29 to 123.66) to 119.29 (95% CI 118.68 to 119.91) and from 54.91 (95% CI 54.52 to 55.29) to 51.04 (95% CI 50.74 to 51.35), respectively.

**Conclusion and implications:**

We observed a spike in antipsychotic prescribing in the dementia and care home groups, which correlated with lockdowns and was likely due to prescribing of antipsychotics for palliative care. We observed gradual increases in antipsychotic use in dementia and care home patients and decreases in their use in patients with learning disability or autism.

**WHAT IS ALREADY KNOWN ON THIS TOPIC**

- Antipsychotics can be prescribed to manage behavioural disturbance in people with dementia or learning disability.
- This should only be considered after non-pharmacological management options have been ruled out.

**WHAT THIS STUDY ADDS**

- This study describes the use of antipsychotics within these at-risk populations during periods where care options were restricted.

**HOW THIS STUDY MIGHT AFFECT RESEARCH, PRACTICE OR POLICY**

- This study demonstrates how the pandemic did not prompt widespread antipsychotic prescribing to manage challenging behaviour; however, some further efforts need to be made to reduce antipsychotic prescribing in dementia.

## Background

In March 2020, the UK went into its first national lockdown following the outbreak of the SARS-CoV-2 pandemic. Subsequent literature has described the negative psychological impact of these lockdowns on ‘at-risk’ groups of patients, such as those with dementia,^1 2^ learning disability^3^ and autism,^4 5^ including increases in agitation, anxiety, depressive symptoms and negative behaviours such as aggressive and self-harming behaviours, which are sometimes treated with antipsychotics.

Prior to the pandemic, the National Health Service (NHS) in England had recognised that prescribing of antipsychotics was excessive in certain at-risk populations including in patients with dementia, learning disability, autism and those in care homes. In response, national campaigns, such as the National Dementia Strategy (2009) and Stopping Over Medication of People with a learning disability, autism or both (2016), were funded to reduce the overprescribing of antipsychotics in these groups.^6 7^ The National Institute for Health and Care Excellence states that in patients with learning disability or dementia, antipsychotics should only be used to manage behaviour where patients are severely distressed or at risk of harming themselves or others^8 9^ and advocates the use of non-pharmacological strategies, including structured activities and positive social support as a preferred approach to managing challenging behaviours. However, many of the social and charitable structures that supported people with disabilities were substantially affected during the pandemic.^6^ We were interested to know if the combination of negative impacts of the pandemic led to an increase in antipsychotic use within these at-risk populations.

OpenSAFELY is a new secure analytics platform for electronic patient records built by our group on behalf of NHS England to deliver urgent academic^10–12^ and operational research during the pandemic.^13–15^ One of the aims of this research platform is to assess the effect of the pandemic on indirect health-related outcomes, such as the impact of the dramatic change in services on at-risk populations. OpenSAFELY analyses can currently run across all patients’ full raw pseudonymised primary care records at 99% of English general practices, with patient-level linkage to various sources of secondary care data. All code and analysis is shared openly for inspection and reuse.

We therefore set out to use the OpenSAFELY platform to assess the impact of the COVID-19 pandemic on antipsychotic prescribing trends in at-risk populations and to establish if there was a change in prescribing practice.

## Methods

### Study design

We conducted a retrospective population-based cohort study using general practice primary care electronic health record (EHR) data accessed through OpenSAFELY, which covered 99% of England’s general practices. Data were included from January 2019 to December 2021 and therefore included 14 months of prescribing prior to the first lockdown in March 2020 and 22 months after this point.

### Data source

Primary care records for all practices in England managed by the general practice (GP) software providers TPP and EMIS are available in OpenSAFELY, a data analytics platform created by our team on behalf of NHS England to address urgent COVID-19 research questions (https://opensafely.org). OpenSAFELY provides a secure software interface allowing the analysis of pseudonymised primary care patient records from England in near real-time within the EHR vendor’s highly secure data centre, avoiding the need for large volumes of potentially disclosive pseudonymised patient data to be transferred off-site. This, in addition to other technical and organisational controls, minimises any risk of re-identification. The TPP dataset analysed within OpenSAFELY (OpenSAFELY-TPP) is based on 24.8 million people currently registered with GP surgeries using TPP SystmOne software; the EMIS dataset analysed within OpenSAFELY (OpenSAFELY-EMIS) is based on 35.2 million people currently registered with GP surgeries using EMIS. It includes pseudonymised data such as coded diagnoses, medications and physiological parameters. No free-text data are included. Further details on our information governance can be found in the information governance and ethics section below.

### Study population

We included all individuals who were alive and registered at an OpenSAFELY-TPP or OpenSAFELY-EMIS practice each month, across the study period.

### At-risk groups

Five at-risk groups were identified consisting of individuals who had a recorded history of a diagnosis of learning disability, autism, dementia, serious mental illness or a history of being a resident in a nursing or residential care home according to their GP record. These groups were non-exclusive (eg. patients in a care home and with dementia were counted in both groups). For the care home group, we included all those coded by GPs as living in residential accommodation, however this is not a comprehensive list of all patients who live in supported accommodation.^16^ For our dementia cohort, we excluded any patient under the age of 35 years, where a diagnosis of dementia is extremely rare. The serious mental illnesses identified include all forms of psychotic illness, schizophrenia and bipolar disorders. Further details can be found under the ‘Codelists and implementation’ section and in the online supplemental material S2.

10.1136/bmjment-2023-300775.supp1 Supplementary data

### Antipsychotic medications

Prescriptions for antipsychotic medications were defined using NHS Dictionary of Medicines and Devices codes (codelist details can be found in the online supplemental material S1). We categorised antipsychotic medication in terms of whether they were first or second generation, or long-acting injectable and depot antipsychotics, and those that are routinely used for other indications, such as prochlorperazine which is used extensively for nausea and vomiting (online supplemental table S3). We excluded antipsychotics which are exclusively used in palliative care, such as the injectable forms of levomepromazine. Over the study period, only the most recently available specified code for a prescription of an antipsychotic medication within each month was included.

### Population characteristics

To summarise the population, we counted all patients registered within the period (1 October to 31 December 2021) and those who received one or more prescriptions for an antipsychotic during this period, and we described the demographics of this subgroup compared with the total population. This was repeated for each at-risk group.

We extracted patient demographic information on age (categorised into eight classes), sex, deprivation, ethnicity and region (seven NHS England regions). Deprivation was measured by the index of multiple deprivation (IMD) in quintiles, with higher values indicating greater deprivation, derived from the patient’s postcode at lower super output area level for a high degree of precision. Ethnicity was collapsed into the 16 census categories of white (including British, Irish, other white), south Asian (Indian, Pakistani, Bangladeshi, other Asian), black (African, Caribbean, other black), other (Chinese, all other) and mixed (white and Asian, white and African, white and Caribbean, other mixed) and unknown.

### Trends in antipsychotic prescribing

We counted the number of patients who were issued an antipsychotic prescription each month between January 2019 and December 2021. We calculated this for the population as a whole and for each of the five at-risk groups. From this, we calculated both a monthly and quarterly rate (per 1000 eligible population) of antipsychotic prescribing for each group and for the population as a whole. The quarterly rate is preferred because there is more variation at a monthly level and there are expected anomalies with prescribing data in certain months (eg. February and December) due to reduced GP working hours within those months. Meanwhile, the monthly data were included so that we could show significant changes in prescribing that were relatively short lived.

### Trends in patients’ newly prescribed antipsychotics

We counted the number of patients who were newly prescribed an antipsychotic (those with no recorded antipsychotic prescription in the 24 months prior) each month between January 2019 and December 2021. We calculated the rate (per 1000 eligible population) of newly initiated patients for the population as a whole and in each at-risk group.

### Sensitivity analysis

We completed a further sensitivity analysis in patients with dementia and in care homes to identify if newly prescribed antipsychotics were being initiated for palliative care purposes. For this analysis, we excluded any patient who had died within 2 weeks of the antipsychotic being initiated (one indicator of palliative care) and any patient who had been prescribed midazolam, a drug commonly used in palliative care, at the same time (±1 day) as the antipsychotic.

### Missing data

Individuals with missing ethnicity, IMD and region were included as ‘unknown’. Individuals were excluded if they had an unknown date of birth or unknown sex.

### Statistical methods

Simple descriptive statistics were used to summarise the number of patients issued antipsychotic prescriptions, the number of first antipsychotic prescriptions and the monthly rate of antipsychotic prescribing. Rates, with associated 95% CIs, were estimated by dividing antipsychotic counts by the total at-risk population and multiplying by 1000. Counts and rates of antipsychotic prescriptions were stratified by each demographic variable, within each of the five at-risk populations.

Charts and results not presented in this manuscript are available online for inspection in the associated GitHub repository.^17^ Patient counts of 0–7 are shown as ‘<8’ with remaining counts rounded to the nearest 10 to protect against small number differences.

### Codelists and implementation

Information on all covariates were obtained from primary care records by searching TPP/EMIS records for specific coded data. Detailed information on compilation and sources for every individual codelist is available at https://www.opencodelists.org/ and the lists are available for inspection and reuse by the broader research community. Links to specific codelists used within this study can be found in the online supplementary material. All codelists were verified and reviewed by contributors from within the Datalab team.

### Software and reproducibility

Data management was performed using the OpenSAFELY software libraries, implemented using Python 3.8, with analysis carried out using both Python and R V.4.0.2. This was an analysis delivered using federated analysis through the OpenSAFELY platform. A federated analysis involves carrying out patient-level analysis in multiple secure datasets, then later combining them: codelists and code for data management and data analysis were specified once using the OpenSAFELY tools; then transmitted securely from the OpenSAFELY jobs server to the OpenSAFELY-TPP platform within TPP’s secure environment, and separately to the OpenSAFELY-EMIS platform within EMIS’s secure environment, where they were each executed separately against local patient data; summary results were then reviewed for disclosiveness, released and combined for the final outputs. All code for the OpenSAFELY platform for data management, analysis and secure code execution is shared for review and reuse under open licences on GitHub: https://github.com/opensafely/antipsychotics-prescribing-during-COVID-19.

### Data sharing and ethics

This study was approved by the Health Research Authority (REC reference 20/LO/0651) and by the LSHTM Ethics Board (reference 21863). NHS England is the data controller of the NHS England OpenSAFELY COVID-19 Service; EMIS and TPP are the data processors. All study authors using OpenSAFELY have the approval of NHS England.^18^ This implementation of OpenSAFELY is hosted within the EMIS and TPP environments which are accredited to the ISO 27001 information security standard and are NHS IG Toolkit compliant.^19^ Patient data has been pseudonymised for analysis and linkage using industry standard cryptographic hashing techniques; all pseudonymised datasets transmitted for linkage onto OpenSAFELY are encrypted. Access to the NHS England OpenSAFELY COVID-19 service is via a virtual private network (VPN) connection; the researchers hold contracts with NHS England and only access the platform to initiate database queries and statistical models; all database activity is logged. Only aggregate statistical outputs leave the platform environment following best practice for anonymisation of results such as statistical disclosure control for low cell counts.^20^ The service adheres to the obligations of the UK General Data Protection Regulation (UK GDPR) and the Data Protection Act 2018. The service previously operated under notices initially issued in February 2020 by the the Secretary of State under Regulation 3(4) of the Health Service (Control of Patient Information) Regulations 2002 (COPI Regulations), which required organisations to process confidential patient information for COVID-19 purposes; this set aside the requirement for patient consent.^21^ As of 1 July 2023, the Secretary of State has requested that NHS England continue to operate the Service under the COVID-19 Directions 2020.^22^ In some cases of data sharing, the common law duty of confidence is met using, for example, patient consent or support from the Health Research Authority Confidentiality Advisory Group.^23^ Taken together, these provide the legal bases to link patient datasets using the service. GP practices, which provide access to the primary care data, are required to share relevant health information to support the public health response to the pandemic, and have been informed of how the service operates.

## Results

### Population characteristics

Between 1 October and 31 December 2021, 59 968 090 individuals were alive and registered at a TPP or EMIS GP practice. Demographics of this cohort are described in table 1. Within this total population, 543 220 (<1%) patients were prescribed an antipsychotic. Rates of antipsychotic prescribing increased with increasing age and with increasing levels of deprivation; 29% of individuals prescribed an antipsychotic were in the most deprived quintile compared with 12% in the least deprived. A higher proportion of those issued an antipsychotic were female (57%); the baseline population had a 50:50 ratio of women to men.

**Table 1:** Demographics of cohort, split by antipsychotic use

Attribute	Category	Combined figures from TPP and EMIS		
		Total (%)	Not on antipsychotic (%)	Taking antipsychotic (%)
Total		59 968 090 (100)	59 424 880 (100)	543 220 (100)
Age group (years)	0–17	11 283 265 (18.8)	11 278 660 (19)	4610 (0.8)
	18–24	4 867 130 (8.1)	4 845 160 (8.2)	21 970 (4)
	25–34	8 647 075 (14.4)	8 584 350 (14.4)	62 730 (11.5)
	35–44	8 543 285 (14.2)	8 462 220 (14.2)	81 065 (14.9)
	45–54	7 766 895 (13)	7 668 135 (12.9)	98 765 (18.2)
	55–69	10 505 365 (17.5)	10 364 955 (17.4)	140 410 (25.8)
	70–79	5 099 270 (8.5)	5 031 145 (8.5)	68 125 (12.5)
	80+	3 255 805 (5.4)	3 190 255 (5.4)	65 545 (12.1)
Sex	Female	29 937 970 (49.9)	29 627 580 (49.9)	310 390 (57.1)
	Male	30 030 130 (50.1)	29 797 300 (50.1)	232 825 (42.9)
IMD	1 Most deprived	12 178 640 (20.3)	12 018 870 (20.2)	159 765 (29.4)
	2	12 376 850 (20.6)	12 251 495 (20.6)	125 350 (23.1)
	3	11 938 615 (19.9)	11 836 940 (19.9)	101 680 (18.7)
	4	11 372 110 (19)	11 290 090 (19)	82 020 (15.1)
	5 Least deprived	11 217 270 (18.7)	11 150 375 (18.8)	66 895 (12.3)
	Unknown	884 615 (1.5)	877 115 (1.5)	7505 (1.4)
Ethnicity	White—British	26 544 735 (44.3)	26 229 235 (44.1)	315 495 (58.1)
	White—Irish	276 660 (0.5)	272 495 (0.5)	4170 (0.8)
	White—any other white background	4 208 045 (7)	4 189 030 (7)	19 010 (3.5)
	Mixed—white and black Caribbean	213 695 (0.4)	211 110 (0.4)	2585 (0.5)
	Mixed—white and black African	196 975 (0.3)	195 685 (0.3)	1290 (0.2)
	Mixed—white and Asian	192 705 (0.3)	191 515 (0.3)	1190 (0.2)
	Mixed—any other mixed background	375 265 (0.6)	372 765 (0.6)	2500 (0.5)
	Asian or Asian British—Indian	1 547 965 (2.6)	1 538 495 (2.6)	9460 (1.7)
	Asian or Asian British—Pakistani	1 206 855 (2)	1 196 145 (2)	10 715 (2)
	Asian or Asian British—Bangladeshi	474 600 (0.8)	470 015 (0.8)	4585 (0.8)
	Asian or Asian British—any other Asian background	975 140 (1.6)	968 960 (1.6)	6175 (1.1)
	Black or black British—Caribbean	375 610 (0.6)	370 040 (0.6)	5570 (1)
	Black or black British—African	1 061 380 (1.8)	1 053 880 (1.8)	7505 (1.4)
	Black or black British—any other black background	270 610 (0.5)	267 710 (0.5)	2895 (0.5)
	Other ethnic groups—any other ethnic group	845 665 (1.4)	840 120 (1.4)	5545 (1)
	Other ethnic groups—Chinese	485 385 (0.8)	484 320 (0.8)	1060 (0.2)
	Unknown	20 716 810 (34.5)	20 573 350 (34.6)	143 455 (26.4)
Region	East of England	7 075 760 (11.8)	7 008 010 (11.8)	67 750 (12.5)
	London	10 123 045 (16.9)	10 048 125 (16.9)	74 920 (13.8)
	Midlands	11 268 450 (18.8)	11 157 880 (18.8)	110 565 (20.4)
	North East and Yorkshire	7 441 485 (12.4)	7 376 235 (12.4)	65 250 (12)
	North West	8 959 840 (14.9)	8 867 515 (14.9)	92 325 (17)
	South East	9 108 570 (15.2)	9 028 185 (15.2)	80 385 (14.8)
	South West	5 956 165 (9.9)	5 904 495 (9.9)	51 670 (9.5)
	Unknown	34 785 (0.1)	34 445 (0.1)	345 (0.1)

IMD, index of multiple deprivation.

### Trends in antipsychotic prescribing

**Figure 1:**
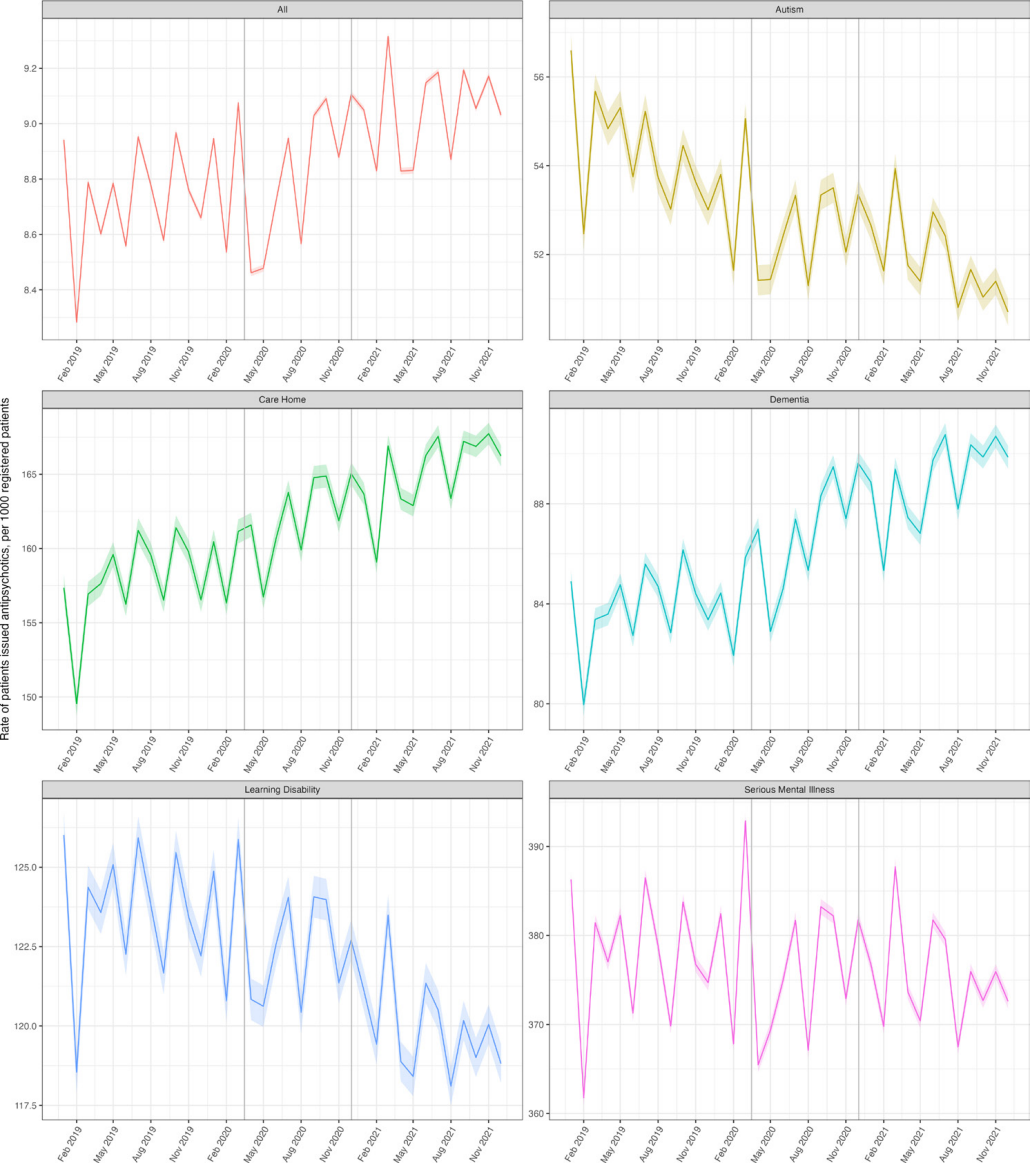
Monthly rate of patients issued an antipsychotic between January 2019 and December 2021, stratified by at-risk group. Solid coloured lines represent the monthly rates for each group with shaded colours areas representing 95% CIs. Vertical grey lines represent the start of the first two national lockdowns. Note each plot has a separate y-axis scale thus plots should be considered separately.

**Figure 2:**
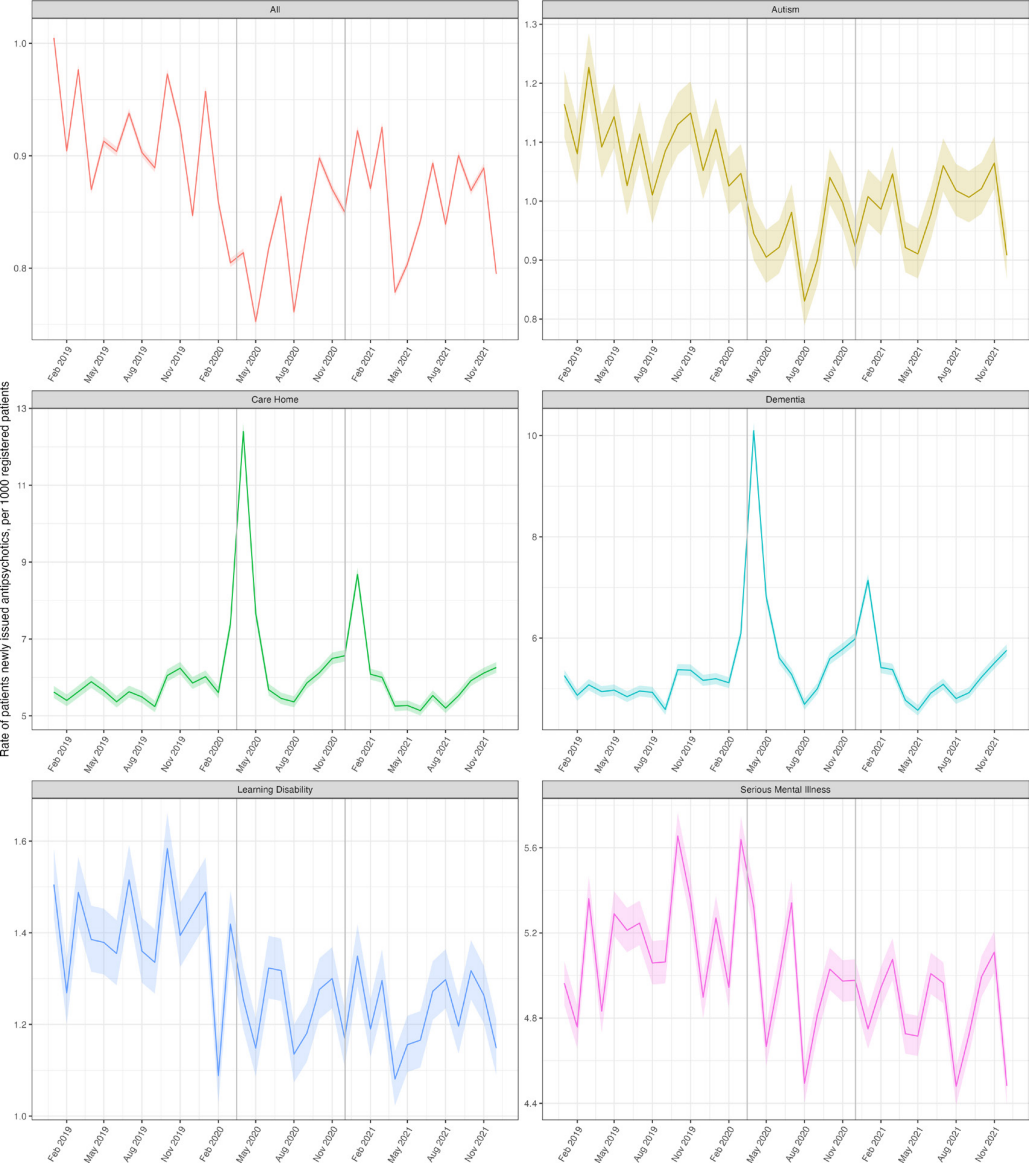
Monthly rate of patients newly issued an antipsychotic between January 2019 and December 2021stratified by at-risk group. Solid coloured lines represent the monthly rates for each group with shaded colours areas representing 95% CIs. Vertical grey lines represent the start of the first two national lockdowns. Note variation in each plot has a separate y-axis scale thus plots should be considered separately.

There was a slight increase in the rate of antipsychotic prescribing (per 1000 patients) over the study period from 8.67 (95% CI 8.66 to 8.68) in January–March 2019 to 9.09 (95% CI 9.07 to 9.10) in October–December 2021 (figure 1). There was a decrease in the overall rate of new initiation of antipsychotics around the time of the first lockdown; in Quarter 2 of 2020 this figure was 0.794 (95% CI 0.791 to 0.799) whereas in Q2 2019 it was 0.895 (95% CI 0.891 to 0.899) (figure 2).

### Demographic and temporal variations in prescribing according to at-risk population

**Figure 3:**
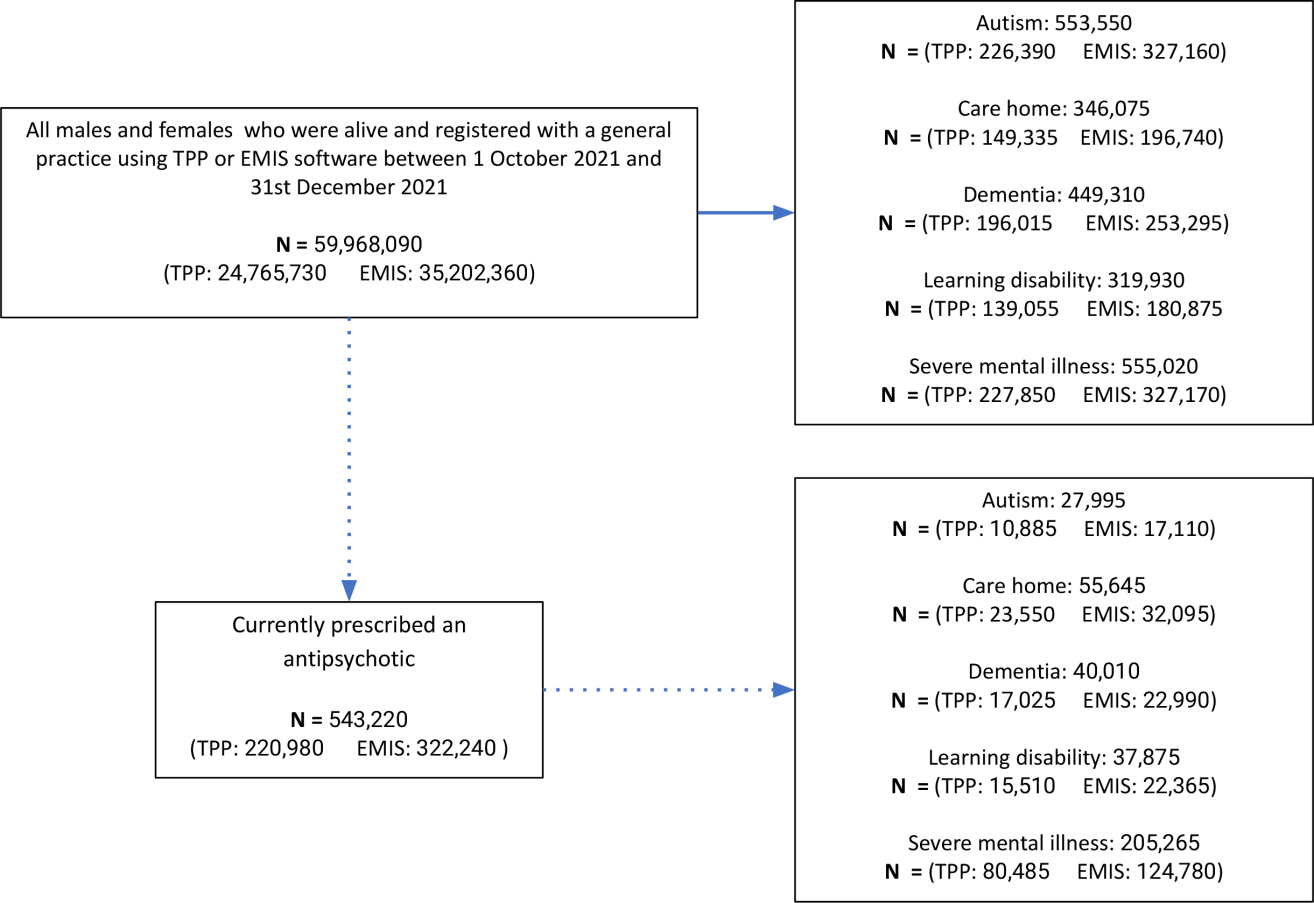
Clinical characteristics.

The populations of each group are shown in figure 3. Further details on demographic and ethnic variabilities within the groups are shown in the online supplemental tables S5–S9.

### Dementia

Between 1 October and 31 December 2021, 449 310 individuals had a record of dementia (figure 3). Within this population, 40 010 (9%) patients were prescribed an antipsychotic. The monthly rate (per 1000 patients) of antipsychotic prescribing increased over the study period from 82.75 (95% CI 82.30 to 83.19) in Q1 (January–March) 2019 to 90.1 (95% CI 89.68 to 90.60) in Q4 (October–December) 2021 (figure 1).

### Care home

Between 1 October and 31 December 2021, 346 075 individuals were classed as residing in a care home (figure 3). Within this population, 55 645 (16%) were prescribed an antipsychotic. The monthly rate (per 1000 patients) of antipsychotic prescribing increased over the study period from 154.61 (95% CI 153.79 to 155.43) in Q1 2019 to 166.95 (95% CI 166.23 to 167.67) in Q4 2021 (figure 1).

### Learning disability

Between 1 October and 31 December 2021, 319 930 individuals were classed as having a learning disability (figure 3). Within this population, 37 875 (12%) were prescribed an antipsychotic. The monthly rate (per 1000 patients) of antipsychotic prescribing decreased over the study period from 122.97 (95% CI 122.29 to 123.66) in Q1 2019 to 119.29 (95% CI 118.68 to 119.91) in Q4 2021 (figure 1).

### Autism

Between 1 October and 31 December 2021, 553 550 individuals had a record of autism (figure 3). From within this population, 27 995 (5%) were prescribed an antipsychotic. The monthly rate (per 1000 patients) of antipsychotic prescribing decreased slightly over the study period from 54.91 (95% CI 54.52 to 55.29) in Q1 2019 to 51.04 (95% CI 50.74 to 51.35) in Q4 2021 (figure 1).

### Serious mental illness

Between 1 October and 31 December 2021, 555 020 individuals had a record of a serious mental illness (figure 3). From within this population, 205 265 (37%) were currently prescribed an antipsychotic. The monthly rate (per 1000 patients) of antipsychotic prescribing within these patients decreased slightly from 376.49 (95% CI 375.60 to 377.38) in Q1 2019 to 373.75 (95% CI 372.92 to 374.57) in Q4 2021 (figure 1).

### Prescribing trends of new prescriptions over time within at-risk populations

#### Dementia and care homes

**Figure 4:**
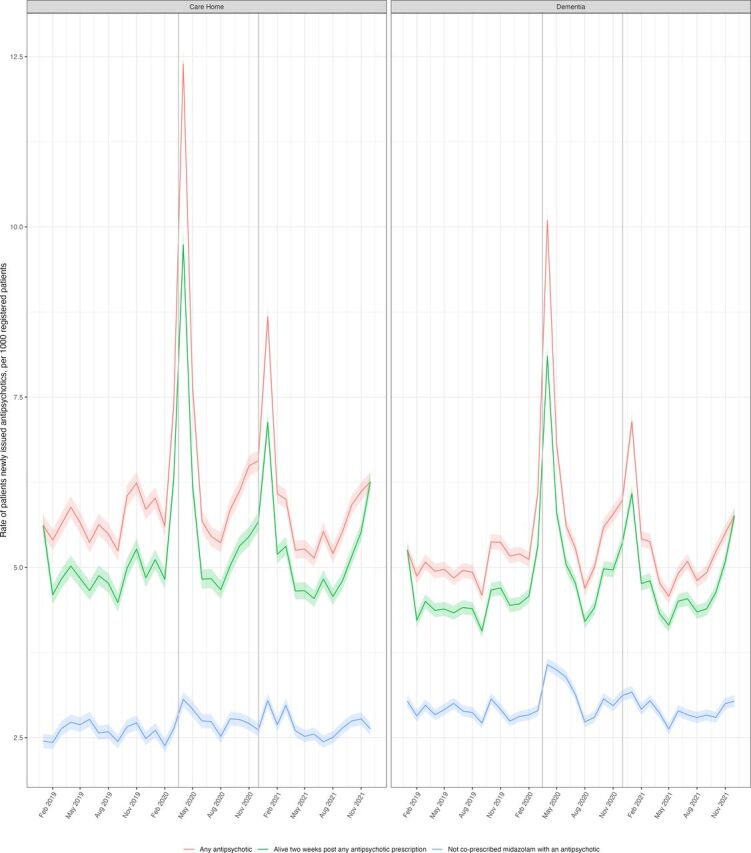
Monthly rates of care home and patients with dementia newly issued an antipsychotic (red line), excluding patients who had died within 2 weeks of being prescribed an antipsychotic (one indicator of palliative care) (green line) and who had also recently been prescribed midazolam (a drug frequently prescribed in palliative care) (blue line).

Over the study period, there were two notable increases in the rate of new antipsychotic prescriptions issued to patients with dementia, which coincided with the start of the COVID-19 pandemic/lockdowns; the rate of new prescriptions issued to patients with dementia rose from 5.26 (95% CI 5.14 to 5.37) in January 2020 to 10.09 (95% CI 9.94 to 10.24) in April 2020 and from 5.98 (95% CI 5.86 to 6.10) in November 2020 to 7.14 (95% CI 7.01 to 7.27) in December 2020 (figure 2). A similar trend was seen in care homes where the rate of new prescriptions rose from 5.62 (95% CI 5.46 to 5.76) in January 2020 to 12.40 (95% CI 12.17 to 12.62) in April 2020 and from 6.56 (95% CI 6.41 to 6.71) in December 2020 to 8.68 (95% CI 8.51 to 8.50) in January 2021 (figure 2). Our sensitivity analysis showed that when patients who were most likely to be on an palliative or end-of-life pathway were excluded, these two peaks were notably reduced (figure 4).

#### Learning disability and autism

The rate of initiating new antipsychotic prescriptions in patients with learning disability and in patients with autism appears to have decreased slightly over the study period, from 1.42 in Q1 2019 (95% CI 1.35 to 1.50) to 1.24 (95% CI 1.18 to 1.31) in Q4 2021 in learning disability and 1.15 (95% CI 1.10 to 1.21) in Q1 2019 to 0.97 (95% CI 0.96 to 1.04) in Q4 2021, which reflects the decrease in overall use.

#### Serious mental illness

The rate of new antipsychotic prescriptions issued to patients with serious mental illnesses remained relatively unchanged; 5.03 (95% CI 4.93 to 0.513) in Q1 2019 and 4.86 (95% CI 4.77 to 4.96) in Q4 2021. Except for a slight increase in the rate of new first-generation antipsychotic prescribing in patients with serious mental illness between March and May 2020, there were no other notable trends during the course of the pandemic.

## Discussion

### Summary

Over the study period, we observed increases in the rate of antipsychotic prescribing in patients with dementia and in care homes. Furthermore, there were notable peaks in the rate of newly prescribed antipsychotics in these two groups which coincided with the beginning of the first and second lockdowns. Many of these new prescriptions are likely to be associated with palliative care indications. Haloperidol is recommended for the management of restlessness, confusion and vomiting during end-of-life care^24^ and pre-emptive prescribing of palliative care medicines, including antipsychotics, was recommended by some organisations for elderly and frail patients in residential care, to allow staff to be ready to use it should the patient become infected with COVID-19.^25^ Results from our sensitivity analysis are consistent with the suggestion that these spikes are due to palliative care prescribing. Conversely, we observed a decreasing trend during the same period in the prescribing of antipsychotics in patients with learning disability or autism, although we found some notable variations in frequency of use associated with ethnicity which requires further investigation. We did not observe an increase in new prescriptions for antipsychotics in the learning disability and autistic population and found no consistent changes in the prescribing trends of antipsychotics in patients with a severe mental illness.

### Strengths and weaknesses

The key strength of this study is its scale; the OpenSAFELY platform runs analyses across the full dataset of all raw, pseudonymised, single-event-level clinical events for 59.9 million patients registered at all NHS GP practices in England using EMIS and TPP software. This federated analytics allows us to explore in detail medication usage, diagnostic events and other salient clinical, regional and demographic information including ethnicity, age and scores of deprivation. Using tailor-made codelists, we were able to design further flexibility into our data extraction which allowed for a more accurate understanding of the prescribing trends.

We also note some limitations. While we have included some prepandemic data in our analysis, this provides a brief reflection of the prepandemic trend and therefore we urge caution in interpreting prescribing trends as being associated with the pandemic.^26^ Furthermore, the data does not elucidate changes in diagnostic or consultation frequency during the study period. Some of the conditions that we were exploring are reported to not be well coded in primary care records.^27^ For example, in April 2021, NHS Digital estimated that only 61.7% of patients with dementia had a recorded code for dementia within their GP notes.^28^ There is therefore some uncertainty about whether the identified population is fully representative of the dementia population as a whole. Furthermore, our previous work has shown that there is currently no canonical data source to identify individuals resident in a care home; and that all methods used for identifying care home residents will likely represent an underestimate; these limitations will apply to all UK healthcare database studies.^16^ All clinical groups were assessed independently, we recognise that there is significant overlap between groups such as our care home population and those with dementia or learning disability. Further analyses to explore the detail of this overlap was outside the scope of this study. We did not observe an increase in new prescriptions within the learning disability or autism cohorts, yet this must be understood within the context of our study definition: a new prescription was defined as ‘an antipsychotic prescription in those without any antipsychotic prescription in the previous 24 months’. This means that if a patient had previously taken an antipsychotic within the 2-year time period, and this antipsychotic was restarted or the dose was increased during lockdown we would not have included this in our count of new prescriptions. As a federated analysis has been carried out across two EHR vendor’s systems, it is possible that a very small number of patient records are duplicated; however, this has now been established to represent <0.03% of the total patient count. It is also possible that we counted some patients twice, if they moved GP practice and changed EHR vendor during the study period. Finally, these data only include antipsychotics which are prescribed in primary care. In our experience, some antipsychotics are prescribed directly from secondary care in some regions^29^; this includes clozapine and, in some areas, the more expensive antipsychotic injections such as Xeplion, Zypadhera, Trevicta and Abilify Maintena. Furthermore, some antipsychotics may be prescribed by secondary care services for acute behavioural disturbance, for example, via psychiatric outreach teams or emergency care services, and this prescribing will not be reflected in our data.

### Findings in context

To our knowledge, this is the first study of its scale that used NHS GP data to describe antipsychotic prescribing trends in different at-risk patient groups, during and after the COVID-19 pandemic.

NHS Digital publishes rates of antipsychotic prescribing in dementia over the previous 12-month period, via an interactive dashboard,^28^ which is updated monthly. In November 2020, researchers using these data observed an increase in the rate of antipsychotic prescribing in dementia in the early months of the pandemic and raised concerns that this could be in response to worsened agitation and psychosis secondary to COVID-19 restrictions.^30^ In December 2021, NHS Digital reported that 9.3% of patients with dementia had been prescribed an antipsychotic over the previous 6 weeks. This is a slightly higher figure than we observed (8.99, 95% CI 8.94 to 9.03), although we counted prescriptions issued within a defined month, rather than over 6 weeks, which may account for the difference. There have also been external factors affecting their data; in October 2021, they removed antipsychotics commonly used in palliative care from their dataset^31^ without applying this change to previous time windows, and therefore perceived reductions in their prescribing rates around this time must be viewed with caution. Throughout our data extraction, we excluded antipsychotics which are used exclusively in palliative care, such as the injectable formulations of levomepromazine, however we have included those which are used for both palliative care and the management of behavioural and psychiatric symptoms of dementia, such as haloperidol.

NHS Digital also publishes data about antipsychotic prescribing in learning disability. These data are calculated from a cohort of patients (56% patient coverage in 2020/21) and is reported annually.^32^ In their 2020/21 report, they calculated that 14.8% of patients with a learning disability were prescribed an antipsychotic and 15.2% in 2019/20. These figures are greater than ours, however they count all patients who had a prescription for an antipsychotic within a 6-month period. We have been able to provide significantly more detail in our analysis by using a bigger dataset and a monthly extraction of data. Finally, in Canada, researchers found statistically significant increases in the use of antipsychotics, benzodiazepines, antidepressants, anticonvulsants and opioids in nursing home residents following the onset of the COVID-19 pandemic, although they noted that these increases were not as pronounced in residents who had dementia. We found no UK-based research studies that described antipsychotic prescribing in care homes over the pandemic.

### Implications for policy and research

This initial descriptive piece of work has shown how we can use the OpenSAFELY platform to facilitate near real-time research of prescribing trends in the context of an evolution of healthcare services following the pandemic. The increase in antipsychotic prescribing in dementia and care home patients may be partly explained by palliative care purposes during the height of the pandemic, however the persistent elevation into the postpandemic period is concerning and warrants further investigation.

A key government report recently highlighted concerns about overprescribing within the NHS, noting that it may disproportionately affect those who are more vulnerable such as the elderly and those with disabilities.^33^ This report called for research on overprescribing, including a specific recommendation to prioritise research into the links between overprescribing, deprivation, ethnicity and inequalities and the impact this has on the health of the population.

### Future research

In 2021, NHS England published their Core20Plus5 document which described their commitment to reduce health inequalities across services.^34^ Some of our data suggest evidence of variation between different ethnic groups in the prescribing of antipsychotics (see online supplemental tables S4–S8), however detailed analysis and discussion is outside the scope of this manuscript. We intend to embark on dedicated work to further explore these findings and understand the extent of variability in prescribing within these vulnerable groups. This will enable better understanding of the impact of the pandemic on these vulnerable populations and add to the evidence that supports work on reducing overprescribing and health inequalities.

Using the OpenSAFELY framework, we can conduct rapid near real-time research into prescribing trends of medicines for almost the entire population of England. We can then focus in detail over a range of key variables, including medication type, diagnosis, demographics and ethnicity. With appropriate permissions and where appropriate support can be obtained from relevant professional bodies, the OpenSAFELY platform is also technically capable of providing audit and feedback information about clinical practice, and changes in clinical practice, at single sites.

### Summary

There is still much to learn about how the pandemic and subsequent lockdowns have affected the mental health of at-risk populations. We have shown that over the course of the pandemic, there have been increases in antipsychotic use in certain at-risk populations, specifically patients with dementia and those who reside in care homes. We have also shown that there were significant brief increases in antipsychotic prescribing in patients with dementia or in those who were resident in care homes during each of the lockdown periods which are likely to be due to palliative care prescribing. Meanwhile, there has been a decrease in antipsychotic prescribing in patients with learning disability or autism. We need to continue to promote and support research and policy development that reduce inappropriate prescribing of antipsychotics within these vulnerable groups of patients.

## Data Availability

Data are available in a public, open access repository. Access to the underlying identifiable and potentially re-identifiable pseudonymised electronic health record data is tightly governed by various legislative and regulatory frameworks and is restricted by best practice. The data in OpenSAFELY are drawn from general practice data across England where TPP and EMIS are the data processors. TPP developers (CB, JC, JP, FH and SH) and EMIS developers (SO'H, AE, RJ, DA, IW and NP) initiate an automated process to create pseudonymised records in the core OpenSAFELY database, which are copies of key structured data tables in the identifiable records. These are linked onto key external data resources that have also been pseudonymised via SHA-512 one-way hashing of NHS numbers using a shared salt. DataLab developers and principal investigators (AG, AW, RC, HC, BB-C, CA, CM, DE, PI, ID, JM, LF, SB, WH and SD) hold contracts with NHS England and have access to the OpenSAFELY pseudonymised data tables as needed to develop the OpenSAFELY tools. These tools in turn enable researchers with OpenSAFELY Data Access Agreements to write and execute code for data management and data analysis without direct access to the underlying raw pseudonymised patient data, and to review the outputs of this code. All codes for the full data management pipeline—from raw data to completed results for this analysis—and for the OpenSAFELY platform as a whole are available for review at https://github.com/OpenSAFELY. The data management and analysis code for this paper was led by AG.
