# Supplementary material for: Impact of the COVID-19 pandemic on antipsychotic prescribing in individuals with autism, dementia, learning disability, serious mental illness or living in a care home: a federated analysis of 59 million patients’ primary care records in situ using OpenSAFELY
Source: BMJ Ment Health. 2023 Sep 15;26(1):e300775. doi: 10.1136/bmjment-2023-300775 (PMC11146375; doi:10.1136/bmjment-2023-300775)

Supplementary material for OpenSAFELY paper:

The impact of the COVID-19 pandemic on Antipsychotic Prescribing in individuals with autism, dementia, learning disability, serious mental illness or living in a care home: A federated analysis of 59 million patients' primary care records in situ using OpenSAFELY.

Codelists

Table S1: Antipsychotics

Antipsychotic medication	Codelists
First generation antipsychotics, excluding long acting depots	https://codelists.opensafely.org/codelist/opensafely/first-generation-antipsychotics-excluding-long-acting-depots-dmd/1e9b227c/
Second generation antipsychotics excluding long acting injections	https://codelists.opensafely.org/codelist/opensafely/second-generation-antipsychotics-excluding-long-acting-injections/6c7c3c11/
Long acting injectable and depot antipsychotics	https://codelists.opensafely.org/codelist/opensafely/long-acting-injectable-and-depot-antipsychotics-dmd/536cc8dc/
Prochlorperazine	https://codelists.opensafely.org/codelist/opensafely/prochlorperazine-dmd/058baf47/

Table S2: At risk groups

At risk group	Codelists
Learning disability	https://www.opencodelists.org/codelist/nhsd-primary-care-domain-refsets/ld_cod/20210127/
Autism	https://codelists.opensafely.org/codelist/nhsd-primary-care-domain-refsets/autism_cod/20210127/
Serious Mental Illness	https://www.opencodelists.org/codelist/nhsd-primary-care-domain-refsets/mh_cod/20210127/
Care homes	https://www.opencodelists.org/codelist/primis-covid19-vacc-uptake/longres/v1/
Dementia	https://www.opencodelists.org/codelist/nhsd-primary-care-domain-refsets/dem_cod/20210127/

Table S3 Antipsychotics included in the study, according to group. Further details about products included within each codelist can be found at https://codelists.opensafely.org/

First Generation Antipsychotics	Second Generation Antipsychotics	Long acting injectable antipsychotics	Prochlorperazine
Benperidol<br>Chlorpromazine<br>Flupentixol<br>Haloperidol<br>Levomepromazine<br>Loxapine<br>Pericyazine<br>Pimozide<br>Promazine<br>Sulpiride<br>Trifluoperazine<br>Zuclopenthixol	Amisulpride<br>Aripiprazole<br>Asenapine<br>Cariprazine<br>Clozapine<br>Lurasidone<br>Olanzapine<br>Paliperidone<br>Quetiapine<br>Risperidone	Aripiprazole<br>Flupentixol decanoate<br>Fluphenazine decanoate<br>Haloperidol decanoate<br>Olanzapine embonate<br>Paliperidone<br>Piportil Depot<br>Risperidone<br>Zuclopenthixol decanoate	Prochlorperazine

Table S4 (See Table 1 in manuscript) Characteristics of patients registered showing detail of TPP or EMIS origin. Note, this data indicates the number of patients who have received a prescription for an antipsychotic, rather than the number of prescriptions issued. Counts are rounded to the nearest 5.

		TPP			EMIS			Combined		
**Attribute**	**Category**	**Total (%)**	**Non-antipsychotic (%)**	**Antipsychotic (%)**	**Total (%)**	**Non-antipsychotic (%)**	**Antipsychotic (%)**	**Total (%)**	**Non-antipsychotic (%)**	**Antipsychotic (%)**
**Total**		24,765,730 (100)	24,544,750 (100)	220,980 (100)	35,202,360 (100)	34,880,130 (100)	322,240 (100)	59968090 (100)	59424880 (100)	543220 (100)
**Age group**	0-17	4,644,690 (18.8)	4,642,945 (18.9)	1,750 (0.8)	6,638,575 (18.9)	6,635,715 (19)	2,860 (0.9)	11,283,265 (18.8)	11,278,660 (19)	4,610 (0.8)
	18-24	1,920,725 (7.8)	1,912,180 (7.8)	8,545 (3.9)	2,946,405 (8.4)	2,932,980 (8.4)	13,425 (4.2)	4,867,130 (8.1)	4,845,160 (8.2)	21,970 (4)
	25-34	3,421,385 (13.8)	3,395,605 (13.8)	25,780 (11.7)	5,225,690 (14.8)	5,188,745 (14.9)	36,950 (11.5)	8,647,075 (14.4)	8,584,350 (14.4)	62,730 (11.5)
	35-44	3,440,625 (13.9)	3,407,630 (13.9)	32,995 (14.9)	5,102,660 (14.5)	5,054,590 (14.5)	48,070 (14.9)	8,543,285 (14.2)	8,462,220 (14.2)	81,065 (14.9)
	45-54	3,214,825 (13)	3,175,245 (12.9)	39,580 (17.9)	4,552,070 (12.9)	4,492,890 (12.9)	59,185 (18.4)	7,766,895 (13)	7,668,135 (12.9)	98,765 (18.2)
	55-69	4,473,325 (18.1)	4,417,190 (18)	56,135 (25.4)	6,032,040 (17.1)	5,947,765 (17.1)	84,275 (26.2)	10,505,365 (17.5)	10,364,955 (17.4)	140,410 (25.8)
	70-79	2,236,870 (9)	2,208,410 (9)	28,460 (12.9)	2,862,400 (8.1)	2,822,735 (8.1)	39,665 (12.3)	5,099,270 (8.5)	5,031,145 (8.5)	68,125 (12.5)
	80+	1,413,285 (5.7)	1,385,545 (5.6)	27,735 (12.6)	1,842,520 (5.2)	1,804,710 (5.2)	37,810 (11.7)	3,255,805 (5.4)	3,190,255 (5.4)	65,545 (12.1)
**Sex**	Female	12,367,160 (49.9)	12,239,700 (49.9)	127,460 (57.7)	17,570,810 (49.9)	17,387,880 (49.9)	182,930 (56.8)	29,937,970 (49.9)	29,627,580 (49.9)	310,390 (57.1)
	Male	12,398,575 (50.1)	12,305,050 (50.1)	93,525 (42.3)	17,631,555 (50.1)	17,492,250 (50.1)	139,300 (43.2)	30,030,130 (50.1)	29,797,300 (50.1)	232,825 (42.9)
**IMD**	1 most deprived	4,923,985 (19.9)	4,862,120 (19.8)	61,860 (28)	7,254,655 (20.6)	7,156,750 (20.5)	97,905 (30.4)	12,178,640 (20.3)	12,018,870 (20.2)	159,765 (29.4)
	2	4,835,865 (19.5)	4,785,965 (19.5)	49,895 (22.6)	7,540,985 (21.4)	7,465,530 (21.4)	75,455 (23.4)	12,376,850 (20.6)	12,251,495 (20.6)	125,350 (23.1)
	3	5,087,635 (20.5)	5,043,835 (20.5)	43,805 (19.8)	6,850,980 (19.5)	6,793,105 (19.5)	57,875 (18)	11,938,615 (19.9)	11,836,940 (19.9)	101,680 (18.7)
	4	4,784,120 (19.3)	4,750,065 (19.4)	34,055 (15.4)	6,587,990 (18.7)	6,540,025 (18.8)	47,965 (14.9)	11,372,110 (19)	11,290,090 (19)	82,020 (15.1)
	5 least deprived	4,375,810 (17.7)	4,350,135 (17.7)	25,675 (11.6)	6,841,460 (19.4)	6,800,240 (19.5)	41,220 (12.8)	11,217,270 (18.7)	11,150,375 (18.8)	66,895 (12.3)
	Unknown	758,320 (3.1)	752,635 (3.1)	5,690 (2.6)	126,295 (0.4)	124,480 (0.4)	1,815 (0.6)	884,615 (1.5)	877,115 (1.5)	7,505 (1.4)
**Ethnicity**	White - British	10,311,070 (41.6)	10,190,620 (41.5)	120,445 (54.5)	16,233,665 (46.1)	16,038,615 (46)	195,050 (60.5)	26,544,735 (44.3)	26,229,235 (44.1)	315,495 (58.1)
	White - Irish	83,845 (0.3)	82,635 (0.3)	1,210 (0.5)	192,815 (0.5)	189,860 (0.5)	2,960 (0.9)	276,660 (0.5)	272,495 (0.5)	4,170 (0.8)
	White - Any other White background	1,512,220 (6.1)	1,506,095 (6.1)	6,125 (2.8)	2,695,825 (7.7)	2,682,935 (7.7)	12,885 (4)	4,208,045 (7)	4,189,030 (7)	19,010 (3.5)
	Mixed - White and Black Caribbean	70,975 (0.3)	70,125 (0.3)	850 (0.4)	142,720 (0.4)	140,985 (0.4)	1,735 (0.5)	213,695 (0.4)	211,110 (0.4)	2,585 (0.5)
	Mixed - White and Black African	66,545 (0.3)	66,105 (0.3)	440 (0.2)	130,430 (0.4)	129,580 (0.4)	850 (0.3)	196,975 (0.3)	195,685 (0.3)	1,290 (0.2)

	Mixed - White and Asian	68,575 (0.3)	68,165 (0.3)	410 (0.2)	124,130 (0.4)	123,350 (0.4)	780 (0.2)	192,705 (0.3)	191,515 (0.3)	1,190 (0.2)
	Mixed - Any other mixed background	108,565 (0.4)	107,830 (0.4)	735 (0.3)	266,700 (0.8)	264,935 (0.8)	1,765 (0.5)	375,265 (0.6)	372,765 (0.6)	2,500 (0.5)
	Asian or Asian British - Indian	566,770 (2.3)	563,250 (2.3)	3,515 (1.6)	981,195 (2.8)	975,245 (2.8)	5,945 (1.8)	1,547,965 (2.6)	1,538,495 (2.6)	9,460 (1.7)
	Asian or Asian British - Pakistani	434,565 (1.8)	430,580 (1.8)	3,990 (1.8)	772,290 (2.2)	765,565 (2.2)	6,725 (2.1)	1,206,855 (2)	1,196,145 (2)	10,715 (2)
	Asian or Asian British - Bangladeshi	99,360 (0.4)	98,510 (0.4)	850 (0.4)	375,240 (1.1)	371,505 (1.1)	3,735 (1.2)	474,600 (0.8)	470,015 (0.8)	4,585 (0.8)
	Asian or Asian British - Any other Asian background	308,105 (1.2)	306,275 (1.2)	1,825 (0.8)	667,035 (1.9)	662,685 (1.9)	4,350 (1.3)	975,140 (1.6)	968,960 (1.6)	6,175 (1.1)
	Black or Black British - Caribbean	84,410 (0.3)	83,120 (0.3)	1,290 (0.6)	291,200 (0.8)	286,920 (0.8)	4,280 (1.3)	375,610 (0.6)	370,040 (0.6)	5,570 (1)
	Black or Black British - African	285,070 (1.2)	283,305 (1.2)	1,770 (0.8)	776,310 (2.2)	770,575 (2.2)	5,735 (1.8)	1,061,380 (1.8)	1,053,880 (1.8)	7,505 (1.4)
	Black or Black British - Any other Black background	68,855 (0.3)	68,215 (0.3)	640 (0.3)	201,755 (0.6)	199,495 (0.6)	2,255 (0.7)	270,610 (0.5)	267,710 (0.5)	2,895 (0.5)
	Other Ethnic Groups - Any other ethnic group	294,655 (1.2)	292,655 (1.2)	2,000 (0.9)	551,010 (1.6)	547,465 (1.6)	3,545 (1.1)	845,665 (1.4)	840,120 (1.4)	5,545 (1)
	Other Ethnic Groups - Chinese	140,635 (0.6)	140,290 (0.6)	340 (0.2)	344,750 (1)	344,030 (1)	720 (0.2)	485,385 (0.8)	484,320 (0.8)	1,060 (0.2)
	Unknown	10,261,515 (41.4)	10,186,970 (41.5)	74,540 (33.7)	10,455,295 (29.7)	10,386,380 (29.8)	68,915 (21.4)	20,716,810 (34.5)	20,573,350 (34.6)	143,455 (26.4)
**Region**	East of England	5,767,745 (23.3)	5,711,595 (23.3)	56,150 (25.4)	1,308,015 (3.7)	1,296,415 (3.7)	11,600 (3.6)	7,075,760 (11.8)	7,008,010 (11.8)	67,750 (12.5)
	London	1,812,115 (7.3)	1,800,535 (7.3)	11,580 (5.2)	8,310,930 (23.6)	8,247,590 (23.6)	63,340 (19.7)	10,123,045 (16.9)	10,048,125 (16.9)	74,920 (13.8)
	Midlands	5,277,845 (21.3)	5,227,650 (21.3)	50,195 (22.7)	5,990,605 (17)	5,930,230 (17)	60,370 (18.7)	11,268,450 (18.8)	11,157,880 (18.8)	110,565 (20.4)
	North East and Yorkshire	4,707,565 (19)	4,666,430 (19)	41,135 (18.6)	2,733,920 (7.8)	2,709,805 (7.8)	24,115 (7.5)	7,441,485 (12.4)	7,376,235 (12.4)	65,250 (12)
	North West	2,115,735 (8.5)	2,098,975 (8.6)	16,760 (7.6)	6,844,105 (19.4)	6,768,540 (19.4)	75,565 (23.5)	8,959,840 (14.9)	8,867,515 (14.9)	92,325 (17)
	South East	1,656,560 (6.7)	1,641,305 (6.7)	15,255 (6.9)	7,452,010 (21.2)	7,386,880 (21.2)	65,130 (20.2)	9,108,570 (15.2)	9,028,185 (15.2)	80,385 (14.8)
	South West	3,393,385 (13.7)	3,363,830 (13.7)	29,560 (13.4)	2,562,780 (7.3)	2,540,665 (7.3)	22,110 (6.9)	5,956,165 (9.9)	5,904,495 (9.9)	51,670 (9.5)
	Unknown	34,785 (0.1)	34,445 (0.1)	345 (0.2)	0 (0)	0 (0)	0 (0)	34,785 (0.1)	34,445 (0.1)	345 (0.1)

Table S5: Counts and rates of dementia patients currently prescribed an antipsychotic between 1st October 2021 and 31st December 2021, stratified by demographic variables.

Attribute	Category	TPP		EMIS		Combined			
		Total (%)	Rate per 1000	Total (%)	Rate per 1000	Total (%)	Rate per 1000	Lower 95% CI	Upper 95% CI
Total		17,030 (8.7)	86.88	22,990 (9.1)	90.76	40,020 (8.9)	89.07	88.20	89.94
Age group	0-17	-	-	-	-	-	-	-	-
	18-24	-	-	-	-	-	-	-	-
	25-34	-	-	-	-	-	-	-	-
	35-44	10 (11.1)	111.11	15 (23.1)	230.77	25 (16.1)	161.29	98.07	224.51
	45-54	105 (14.8)	147.89	160 (20.3)	202.53	265 (17.7)	176.67	155.40	197.94
	55-69	1,525 (14.4)	144.28	2,245 (16.5)	165.44	3,770 (15.6)	156.17	151.19	161.16
	70-79	4,690 (11.4)	113.79	6,370 (12.2)	122.19	11,060 (11.8)	118.49	116.28	120.69
	80+	10,700 (7.5)	74.60	14,195 (7.6)	76.02	24,895 (7.5)	75.40	74.47	76.34
Sex	Female	10,540 (8.6)	85.86	14,310 (9)	90.04	24,850 (8.8)	88.22	87.12	89.31
	Male	6,485 (8.9)	88.53	8,675 (9.2)	91.93	15,160 (9)	90.44	89.00	91.88
IMD	1 most deprived	3,465 (10.2)	101.97	4,635 (10.3)	103.33	8,100 (10.3)	102.75	100.51	104.98
	2	3,485 (9.4)	94.06	4,540 (9.5)	95.24	8,025 (9.5)	94.72	92.65	96.80
	3	3,800 (8.8)	88.28	4,720 (9.3)	93.29	8,520 (9.1)	90.99	89.05	92.92
	4	3,115 (7.6)	75.99	4,685 (8.7)	86.85	7,800 (8.2)	82.16	80.34	83.98
	5 least deprived	2,800 (7.5)	74.96	4,345 (7.8)	78.22	7,145 (7.7)	76.91	75.12	78.69
	Unknown	360 (10)	100.14	65 (9.6)	95.59	425 (9.9)	99.42	89.96	108.87
Ethnicity	White - British	9,320 (9)	90.33	15,095 (9.5)	94.56	24,415 (9.3)	92.90	91.73	94.06
	White - Irish	145 (10)	100.35	335 (10.6)	105.85	480 (10.4)	104.12	94.81	113.44
	White - Any other White background	285 (8.4)	84.32	610 (8.8)	87.77	895 (8.7)	86.64	80.96	92.32
	Mixed - White and Black Caribbean	25 (8.5)	84.75	60 (10.3)	102.56	85 (9.7)	96.59	76.06	117.12
	Mixed - White and Black African	15 (17.6)	176.47	20 (9.8)	97.56	35 (12.1)	120.69	80.71	160.67
	Mixed - White and Asian	10 (9.1)	90.91	20 (11.4)	114.29	30 (10.5)	105.26	67.60	142.93
	Mixed - Any other mixed background	15 (9.4)	93.75	40 (9.5)	95.24	55 (9.5)	94.83	69.77	119.89

	Asian or Asian British - Indian	150 (7.1)	70.75	345 (9.3)	92.74	495 (8.5)	84.76	77.29	92.23
	Asian or Asian British - Pakistani	125 (9.3)	92.94	200 (9.4)	94.34	325 (9.4)	93.80	83.60	103.99
	Asian or Asian British - Bangladeshi	25 (8.9)	89.29	100 (9.9)	98.52	125 (9.7)	96.53	79.60	113.45
	Asian or Asian British - Any other Asian background	50 (7.2)	72.46	135 (7.9)	78.72	185 (7.7)	76.92	65.84	88.01
	Black or Black British - Caribbean	100 (8.6)	86.21	335 (8.3)	83.44	435 (8.4)	84.06	76.16	91.96
	Black or Black British - African	35 (7.5)	75.27	145 (8.9)	88.69	180 (8.6)	85.71	73.19	98.24
	Black or Black British - Any other Black background	15 (9.1)	90.91	55 (10.2)	101.85	70 (9.9)	99.29	76.03	122.55
	Other Ethnic Groups - Any other ethnic group	55 (9.1)	90.91	130 (11.9)	119.27	185 (10.9)	109.14	93.42	124.87
	Other Ethnic Groups - Chinese	15 (8.6)	85.71	30 (7.1)	70.59	45 (7.5)	75.00	53.09	96.91
	Unknown	6,640 (8.3)	82.62	5,335 (8.1)	80.97	11,975 (8.2)	81.88	80.41	83.35
**Region**	East of England	4,500 (10.2)	102.03	1,165 (11.3)	113.38	5,665 (10.4)	104.17	101.46	106.89
	London	445 (6.1)	61.21	3,495 (8.5)	84.86	3,940 (8.1)	81.31	78.77	83.85
	Midlands	3,940 (9.4)	93.89	4,720 (10.4)	104.41	8,660 (9.9)	99.35	97.25	101.44
	North East and Yorkshire	3,305 (8.8)	88.10	1,630 (7.6)	76.45	4,935 (8.4)	83.88	81.54	86.22
	North West	1,220 (6.4)	64.35	4,585 (8.5)	84.93	5,805 (8)	79.58	77.53	81.63
	South East	1,165 (7.8)	78.14	5,525 (9.3)	92.66	6,690 (9)	89.76	87.61	91.91
	South West	2,430 (7.8)	78.10	1,865 (8.6)	85.94	4,295 (8.1)	81.32	78.89	83.75
	Unknown	20 (11.1)	111.11	-	-	20 (11.1)	111.11	62.42	159.81

Table S6: Counts and rates of care home patients currently prescribed an antipsychotic between 1st October 2021 and 31st December 2021, stratified by demographic variables.

Attribute	Category	TPP		EMIS		Combined			
		Total (%)	Rate per 1000	Total (%)	Rate per 1000	Total (%)	Rate per 1000	Lower 95% CI	Upper 95% CI
**Total**		23,550 (15.8)	157.70	32,080 (16.3)	163.06	55,630 (16.1)	160.75	159.41	162.08
**Age group**	0-17	30 (7.4)	74.07	30 (4.5)	45.45	60 (5.6)	56.34	42.08	70.59
	18-24	350 (19.2)	191.78	375 (19.4)	194.30	725 (19.3)	193.08	179.02	207.13
	25-34	1,150 (23.2)	231.85	1,310 (25.1)	251.20	2,460 (24.2)	241.77	232.22	251.32
	35-44	1,355 (25.9)	259.33	1,715 (29.6)	296.46	3,070 (27.9)	278.84	268.97	288.70
	45-54	1,945 (28.6)	285.82	2,525 (31.5)	314.64	4,470 (30.1)	301.42	292.58	310.25
	55-69	4,825 (28.6)	286.10	6,780 (30.9)	308.60	11,605 (29.9)	298.83	293.39	304.27
	70-79	4,760 (21.6)	216.27	6,945 (23.5)	234.75	11,705 (22.7)	226.86	222.75	230.97
	80+	9,135 (10)	100.12	12,405 (10)	100.39	21,540 (10)	100.27	98.94	101.61
**Sex**	Female	12,815 (13.5)	135.48	17,100 (13.8)	138.05	29,915 (13.7)	136.94	135.38	138.49
	Male	10,735 (19.6)	196.09	14,995 (20.6)	205.78	25,730 (20.2)	201.62	199.16	204.09
**IMD**	1 most deprived	5,340 (18.1)	180.71	7,205 (18.5)	185.05	12,545 (18.3)	183.18	179.97	186.38
	2	5,350 (17)	169.55	7,050 (17.8)	178.03	12,400 (17.4)	174.27	171.20	177.33
	3	5,325 (16.2)	161.85	6,795 (16.9)	169.49	12,120 (16.6)	166.05	163.09	169.01
	4	4,055 (14)	140.14	5,920 (14.6)	145.60	9,975 (14.3)	143.33	140.52	146.14
	5 least deprived	3,030 (12.8)	127.77	5,000 (13.6)	135.83	8,030 (13.3)	132.67	129.77	135.57
	Unknown	450 (16.8)	167.91	125 (19.4)	193.80	575 (17.3)	172.93	158.80	187.07
**Ethnicity**	White - British	14,325 (16.9)	168.91	22,125 (17.3)	173.24	36,450 (17.2)	171.51	169.75	173.27
	White - Irish	135 (15.8)	157.89	345 (17.9)	178.76	480 (17.2)	172.35	156.93	187.77
	White - Any other White background	365 (14)	140.38	730 (16.3)	163.31	1,095 (15.5)	154.88	145.71	164.05
	Mixed - White and Black Caribbean	65 (27.7)	276.60	115 (25.3)	252.75	180 (26.1)	260.87	222.76	298.98
	Mixed - White and Black African	25 (22.7)	227.27	40 (22.2)	222.22	65 (22.4)	224.14	169.65	278.63
	Mixed - White and Asian	20 (18.2)	181.82	35 (20.6)	205.88	55 (19.6)	196.43	144.52	248.34
	Mixed - Any other mixed background	45 (25.7)	257.14	80 (20.5)	205.13	125 (22.1)	221.24	182.45	260.02
	Asian or Asian British - Indian	175 (20.7)	207.10	340 (22.8)	228.19	515 (22.1)	220.56	201.51	239.61
	Asian or Asian British - Pakistani	115 (27.1)	270.59	140 (26.2)	261.68	255 (26.6)	265.62	233.02	298.23
	Asian or Asian British - Bangladeshi	15 (15.8)	157.89	100 (24.4)	243.90	115 (22.8)	227.72	186.10	269.34
	Asian or Asian British - Any other Asian background	90 (21.2)	211.76	200 (20.8)	208.33	290 (20.9)	209.39	185.29	233.49
	Black or Black British - Caribbean	150 (25.4)	254.24	525 (27.3)	272.73	675 (26.8)	268.39	248.14	288.64

	Black or Black British - African	70 (18.2)	181.82	280 (23.8)	238.30	350 (22.4)	224.36	200.85	247.86
	Black or Black British - Any other Black background	45 (23.1)	230.77	220 (30.8)	307.69	265 (29.1)	291.21	256.15	326.27
	Other Ethnic Groups - Any other ethnic group	85 (18.9)	188.89	180 (20.5)	204.55	265 (19.9)	199.25	175.26	223.24
	Other Ethnic Groups - Chinese	20 (16.7)	166.67	45 (16.1)	160.71	65 (16.2)	162.50	123.00	202.00
	Unknown	7,805 (13.7)	137.17	6,580 (12.4)	124.00	14,385 (13.1)	130.81	128.68	132.95
**Region**	East of England	5,550 (17)	170.27	1,485 (17.1)	171.38	7,035 (17.1)	170.50	166.52	174.49
	London	555 (15.5)	155.46	5,150 (19.2)	191.56	5,705 (18.7)	187.33	182.46	192.19
	Midlands	5,730 (17.6)	176.06	6,215 (17.7)	177.07	11,945 (17.7)	176.58	173.42	179.75
	North East and Yorkshire	4,705 (16.2)	161.66	2,625 (15.6)	155.60	7,330 (15.9)	159.43	155.78	163.08
	North West	1,830 (12)	120.04	6,640 (15)	150.40	8,470 (14.3)	142.60	139.57	145.64
	South East	1,755 (14.6)	145.95	7,275 (15.4)	154.02	9,030 (15.2)	152.38	149.24	155.52
	South West	3,385 (14.1)	140.60	2,705 (15.2)	151.67	6,090 (14.5)	145.31	141.66	148.96
	Unknown	35 (20)	200.00	0 (NaN)	NA	35 (20)	200.00	133.74	266.26

Table S7: Counts and rates of patients with a learning disability currently prescribed an antipsychotic between 1st October 2021 and 31st December 2021, stratified by demographic variables.

Attribute	Category	TPP		EMIS		Combined			
		Total (%)	Rate per 1000	Total (%)	Rate per 1000	Total (%)	Rate per 1000	Lower 95% CI	Upper 95% CI
Total		15,505 (11.2)	111.50	22,365 (12.4)	123.65	37,870 (11.8)	118.37	117.18	119.56
Age group	0-17	220 (1.2)	11.66	330 (1.2)	12.46	550 (1.2)	12.13	11.11	13.14
	18-24	1,225 (6)	59.52	1,820 (6.9)	68.67	3,045 (6.5)	64.67	62.37	66.97
	25-34	2,945 (9.5)	95.40	4,230 (10.9)	108.66	7,175 (10.3)	102.79	100.42	105.17
	35-44	2,635 (12.6)	125.87	3,665 (13.8)	138.35	6,300 (13.3)	132.84	129.56	136.12
	45-54	2,860 (16.2)	162.36	3,975 (17.9)	178.53	6,835 (17.1)	171.39	167.33	175.45
	55-69	4,305 (18.8)	188.16	6,255 (20.8)	207.91	10,560 (19.9)	199.38	195.57	203.18
	70-79	1,110 (19.1)	190.72	1,720 (21.5)	215.13	2,830 (20.5)	204.85	197.30	212.40
	80+	205 (13.8)	137.58	375 (17.8)	177.73	580 (16.1)	161.11	148.00	174.22
Sex	Female	5,860 (10.7)	107.15	8,340 (11.9)	118.74	14,200 (11.4)	113.67	111.80	115.54
	Male	9,650 (11.4)	114.38	14,025 (12.7)	126.76	23,675 (12.1)	121.41	119.86	122.95
IMD	1 most deprived	4,265 (10.4)	104.46	6,355 (11.8)	118.02	10,620 (11.2)	112.17	110.04	114.31
	2	3,820 (12)	120.41	5,480 (12.7)	127.25	9,300 (12.4)	124.35	121.82	126.88
	3	3,280 (12)	120.17	4,450 (13.2)	131.81	7,730 (12.7)	126.61	123.78	129.43
	4	2,400 (11.4)	114.45	3,470 (12.7)	126.60	5,870 (12.1)	121.33	118.23	124.43
	5 least deprived	1,435 (9.5)	94.91	2,530 (11.5)	114.69	3,965 (10.7)	106.64	103.32	109.96
	Unknown	310 (10)	99.52	80 (10.9)	108.84	390 (10.1)	101.30	91.25	111.35
Ethnicity	White - British	9,840 (12.7)	127.49	15,325 (13.9)	139.14	25,165 (13.4)	134.34	132.68	136.00
	White - Irish	50 (16.7)	166.67	150 (18.3)	182.93	200 (17.9)	178.57	153.82	203.32
	White - Any other White background	240 (9.2)	91.95	600 (11.5)	114.72	840 (10.7)	107.14	99.90	114.39
	Mixed - White and Black Caribbean	70 (13.3)	133.33	145 (13.4)	133.64	215 (13.4)	133.54	115.69	151.39
	Mixed - White and Black African	20 (8.2)	81.63	55 (9.6)	95.65	75 (9.1)	91.46	70.76	112.16
	Mixed - White and Asian	35 (12.3)	122.81	55 (10.6)	105.77	90 (11.2)	111.80	88.70	134.90
	Mixed - Any other mixed background	45 (8.3)	82.57	110 (9.1)	90.53	155 (8.8)	88.07	74.20	101.93
	Asian or Asian British - Indian	225 (11.7)	116.58	385 (12.1)	121.45	610 (12)	119.61	110.12	129.10
	Asian or Asian British - Pakistani	315 (8.7)	87.02	485 (8.6)	86.45	800 (8.7)	86.67	80.67	92.68
	Asian or Asian British - Bangladeshi	60 (10.3)	102.56	250 (10.6)	105.71	310 (10.5)	105.08	93.39	116.78
	Asian or Asian British - Any other Asian background	90 (9.2)	92.31	235 (10.5)	105.38	325 (10.1)	101.40	90.38	112.43

	Black or Black British - Caribbean	105 (15.6)	155.56	400 (17)	169.85	505 (16.7)	166.67	152.13	181.20
	Black or Black British - African	80 (8.3)	82.90	355 (9.6)	95.82	435 (9.3)	93.15	84.39	101.90
	Black or Black British - Any other Black background	55 (13.6)	135.80	255 (13.9)	138.96	310 (13.8)	138.39	122.99	153.80
	Other Ethnic Groups - Any other ethnic group	70 (9.6)	95.89	120 (8.4)	83.62	190 (8.8)	87.76	75.28	100.24
	Other Ethnic Groups - Chinese	20 (12.1)	121.21	35 (9.2)	92.11	55 (10.1)	100.92	74.25	127.59
	Unknown	4,185 (8.8)	88.45	3,405 (8.9)	89.11	7,590 (8.9)	88.75	86.75	90.74
**Region**	East of England	3,555 (12.2)	121.79	825 (12.9)	129.41	4,380 (12.3)	123.15	119.51	126.80
	London	530 (9.6)	95.58	4,225 (11.8)	118.10	4,755 (11.5)	115.08	111.81	118.35
	Midlands	3,785 (12.4)	124.42	4,320 (13.5)	134.98	8,105 (13)	129.84	127.01	132.66
	North East and Yorkshire	3,260 (10.7)	107.22	2,130 (12.6)	125.78	5,390 (11.4)	113.86	110.82	116.90
	North West	1,175 (8.3)	82.57	4,750 (12)	120.13	5,925 (11)	110.19	107.39	113.00
	South East	995 (10.5)	105.46	4,545 (12.5)	125.12	5,540 (12.1)	121.07	117.88	124.25
	South West	2,190 (11.1)	111.39	1,570 (11.3)	112.79	3,760 (11.2)	111.97	108.39	115.55
	Unknown	25 (14.3)	142.86	0 (NaN)	NA	25 (14.3)	142.86	86.86	198.86

Table S8: Counts and rates of patients with autism currently prescribed an antipsychotic between 1st October 2021 and 31st December 2021, stratified by demographic variables

**Attribute**	**Category**	**TPP**	**EMIS**	**Combined**

		Total (%)	Rate per 1000	Total (%)	Rate per 1000	Total (%)	Rate per 1000	Lower 95% CI	Upper 95% CI
**Total**		10,885 (4.8)	48.08	17,110 (5.2)	52.30	27,995 (5.1)	50.57	49.98	51.17
**Age group**	0-17	585 (0.6)	5.57	1,055 (0.7)	6.84	1,640 (0.6)	6.32	6.02	6.63
	18-24	2,065 (4)	39.77	3,455 (4.6)	45.75	5,520 (4.3)	43.32	42.17	44.46
	25-34	3,275 (8)	80.22	5,035 (8.9)	89.50	8,310 (8.6)	85.60	83.75	87.44
	35-44	1,910 (14.3)	142.80	2,825 (15.2)	151.68	4,735 (14.8)	147.97	143.75	152.18
	45-54	1,445 (18.3)	183.26	2,185 (19.6)	195.70	3,630 (19.1)	190.55	184.35	196.75
	55-69	1,400 (21.8)	218.24	2,175 (22.4)	224.46	3,575 (22.2)	221.98	214.70	229.26
	70-79	180 (22.4)	223.60	330 (24.8)	248.12	510 (23.9)	238.88	218.14	259.61
	80+	25 (17.2)	172.41	55 (22)	220.00	80 (20.3)	202.53	158.15	246.91
**Sex**	Female	3,225 (5.6)	55.63	5,130 (6)	60.20	8,355 (5.8)	58.35	57.10	59.60
	Male	7,660 (4.5)	45.48	11,980 (5)	49.52	19,640 (4.8)	47.86	47.19	48.53
**IMD**	1 most deprived	2,565 (4.6)	45.60	4,455 (5.2)	52.50	7,020 (5)	49.75	48.58	50.91
	2	2,605 (5.4)	53.88	4,005 (5.5)	55.11	6,610 (5.5)	54.62	53.30	55.93
	3	2,305 (5.1)	50.89	3,395 (5.6)	55.55	5,700 (5.4)	53.57	52.18	54.96
	4	1,860 (4.9)	48.63	2,855 (5.2)	52.46	4,715 (5.1)	50.88	49.43	52.33
	5 least deprived	1,290 (4)	40.46	2,325 (4.4)	44.10	3,615 (4.3)	42.73	41.34	44.12
	Unknown	255 (4)	40.00	75 (5.5)	55.15	330 (4.3)	42.66	38.06	47.27
**Ethnicity**	White - British	6,245 (5.9)	59.01	10,890 (6.4)	63.98	17,135 (6.2)	62.08	61.15	63.00
	White - Irish	25 (6.7)	66.67	80 (9.6)	96.39	105 (8.7)	87.14	70.47	103.80
	White - Any other White background	190 (3.7)	37.11	480 (4.7)	46.69	670 (4.4)	43.51	40.21	46.80
	Mixed - White and Black Caribbean	45 (3.7)	37.19	125 (5)	49.50	170 (4.6)	45.52	38.67	52.36
	Mixed - White and Black African	25 (3.9)	39.06	65 (4.5)	44.98	90 (4.3)	43.17	34.25	52.08
	Mixed - White and Asian	30 (4)	40.27	60 (4.2)	41.52	90 (4.1)	41.10	32.61	49.59
	Mixed - Any other mixed background	45 (3.4)	34.48	105 (3.4)	33.98	150 (3.4)	34.13	28.67	39.59
	Asian or Asian British - Indian	110 (6.3)	63.22	220 (5.9)	59.06	330 (6)	60.38	53.87	66.90
	Asian or Asian British - Pakistani	125 (5.1)	50.92	275 (5.1)	51.35	400 (5.1)	51.22	46.20	56.24
	Asian or Asian British - Bangladeshi	35 (4.7)	46.98	155 (4.8)	47.62	190 (4.8)	47.50	40.75	54.25
	Asian or Asian British - Any other Asian background	70 (5.7)	56.68	160 (4.8)	47.76	230 (5)	50.16	43.68	56.65
	Black or Black British - Caribbean	65 (10.5)	104.84	205 (8.3)	83.00	270 (8.7)	87.38	76.96	97.80
	Black or Black British - African	60 (3.6)	36.47	265 (4.2)	41.54	325 (4)	40.50	36.10	44.90

	Black or Black British - Any other Black background	45 (7.1)	71.43	195 (7.1)	71.04	240 (7.1)	71.11	62.11	80.11
	Other Ethnic Groups - Any other ethnic group	55 (4.3)	43.14	110 (3.9)	39.22	165 (4)	40.44	34.27	46.61
	Other Ethnic Groups - Chinese	20 (5.5)	54.79	35 (4.2)	42.42	55 (4.6)	46.22	34.00	58.43
	Unknown	3,690 (3.7)	36.74	3,685 (3.5)	34.62	7,375 (3.6)	35.65	34.84	36.46
**Region**	East of England	2,590 (5.3)	53.05	595 (5.2)	52.08	3,185 (5.3)	52.86	51.03	54.70
	London	330 (3.8)	37.54	2,935 (4.8)	47.81	3,265 (4.7)	46.53	44.93	48.12
	Midlands	2,915 (5.2)	52.26	3,815 (6.7)	66.97	6,730 (6)	59.69	58.27	61.12
	North East and Yorkshire	1,855 (4.5)	45.20	1,205 (4.6)	45.62	3,060 (4.5)	45.36	43.76	46.97
	North West	635 (3.1)	31.30	3,340 (5.1)	51.23	3,975 (4.7)	46.50	45.06	47.95
	South East	865 (5.4)	53.73	4,030 (4.8)	48.04	4,895 (4.9)	48.95	47.58	50.33
	South West	1,685 (4.8)	47.81	1,190 (5.4)	54.36	2,875 (5)	50.32	48.48	52.16
	Unknown	10 (3.1)	30.77	0 (NaN)	NA	10 (3.1)	30.77	11.70	49.84

Table S9: Counts and rates of patients with a severe mental illness currently prescribed an antipsychotic between 1st October 2021 and 31st December 2021, stratified by demographic variables

**Attribute**	**Category**	**TPP**		**EMIS**		**Combined**			
		**Total (%)**	**Rate per 1000**	**Total (%)**	**Rate per 1000**	**Total (%)**	**Rate per 1000**	**Lower 95% CI**	**Upper 95% CI**
**Total**		80,485 (35.3)	353.24	124,780 (38.1)	381.39	205,265 (37)	369.84	368.24	371.44
**Age group**	0-17	55 (21.6)	215.69	90 (20.5)	204.55	145 (20.9)	208.63	174.67	242.59
	18-24	1,615 (26.8)	268.27	2,765 (28)	280.14	4,380 (27.6)	275.65	267.48	283.81

	25-34	8,080 (27.8)	278.43	12,735 (28.9)	288.78	20,815 (28.5)	284.67	280.80	288.54
	35-44	13,675 (32.4)	324.24	21,230 (34.4)	344.42	34,905 (33.6)	336.22	332.70	339.75
	45-54	17,700 (37.1)	371.22	27,760 (40.4)	404.22	45,460 (39.1)	390.70	387.11	394.29
	55-69	25,000 (39.3)	392.83	39,075 (43.2)	432.15	64,075 (41.6)	415.91	412.69	419.13
	70-79	9,465 (37.1)	370.89	13,965 (41.3)	412.92	23,430 (39.5)	394.84	389.79	399.90
	80+	4,895 (36.2)	361.65	7,170 (39.4)	393.96	12,065 (38)	380.18	373.40	386.96
**Sex**	Female	41,725 (36.7)	367.41	63,525 (39.5)	395.47	105,250 (38.4)	383.85	381.53	386.17
	Male	38,760 (33.9)	339.15	61,255 (36.8)	367.81	100,015 (35.6)	356.15	353.94	358.35
**IMD**	1 most deprived	24,820 (37.7)	376.57	41,315 (41.5)	414.96	66,135 (40)	399.67	396.62	402.71
	2	19,000 (36.3)	362.53	31,720 (38.7)	386.62	50,720 (37.7)	377.23	373.94	380.51
	3	15,455 (34.7)	347.30	21,280 (36.6)	365.89	36,735 (35.8)	357.83	354.17	361.49
	4	11,400 (33.4)	333.58	16,540 (35.6)	356.31	27,940 (34.7)	346.67	342.61	350.74
	5 least deprived	7,975 (31.2)	311.71	13,160 (33.7)	337.00	21,135 (32.7)	326.99	322.58	331.40
	Unknown	1,835 (34.8)	348.20	765 (39.6)	396.37	2,600 (36.1)	361.11	347.23	374.99
**Ethnicity**	White - British	44,300 (36.3)	362.58	73,980 (40.1)	401.27	118,280 (38.6)	385.85	383.65	388.05
	White - Irish	520 (36.6)	366.20	1,255 (39.7)	397.15	1,775 (38.8)	387.55	369.53	405.58
	White - Any other White background	2,705 (30.3)	303.08	5,760 (32.9)	329.43	8,465 (32.1)	320.52	313.69	327.35
	Mixed - White and Black Caribbean	440 (31.8)	317.69	940 (34.6)	346.22	1,380 (33.7)	336.59	318.83	354.34
	Mixed - White and Black African	255 (32.7)	326.92	500 (32.4)	323.62	755 (32.5)	324.73	301.57	347.89
	Mixed - White and Asian	195 (28.3)	282.61	360 (35.3)	352.94	555 (32.5)	324.56	297.56	351.56
	Mixed - Any other mixed background	345 (28.9)	288.70	900 (32.8)	327.87	1,245 (31.6)	315.99	298.44	333.54
	Asian or Asian British - Indian	1,885 (39.5)	394.76	3,085 (40.8)	408.34	4,970 (40.3)	403.08	391.88	414.29
	Asian or Asian British - Pakistani	2,075 (40.4)	403.70	3,465 (43.2)	432.04	5,540 (42.1)	420.97	409.89	432.06
	Asian or Asian British - Bangladeshi	465 (40.6)	406.11	2,140 (45.9)	459.23	2,605 (44.9)	448.75	431.52	465.98
	Asian or Asian British - Any other Asian background	900 (35.1)	350.88	2,180 (39.1)	390.68	3,080 (37.8)	378.15	364.79	391.50
	Black or Black British - Caribbean	820 (32.6)	326.04	2,770 (35.2)	352.42	3,590 (34.6)	346.02	334.71	357.34
	Black or Black British - African	1,140 (31.9)	319.33	3,760 (33.7)	336.92	4,900 (33.3)	332.65	323.34	341.97
	Black or Black British - Any other Black background	395 (29.3)	292.59	1,510 (34.6)	345.93	1,905 (33.3)	333.33	318.36	348.30
	Other Ethnic Groups - Any other ethnic group	870 (32.8)	327.68	1,495 (35.8)	357.66	2,365 (34.6)	346.01	332.07	359.96
	Other Ethnic Groups - Chinese	190 (35.5)	355.14	385 (31.7)	316.87	575 (32.9)	328.57	301.72	355.43

	Unknown	22,995 (34.3)	343.08	20,295 (34.1)	340.86	43,290 (34.2)	342.04	338.82	345.26
**Region**	East of England	18,220 (36.8)	367.75	3,680 (36.2)	362.03	21,900 (36.7)	366.77	361.92	371.63
	London	6,335 (30.6)	305.96	31,115 (34.9)	348.94	37,450 (34.1)	340.84	337.39	344.29
	Midlands	17,585 (37.5)	375.27	19,745 (38.7)	387.01	37,330 (38.1)	381.39	377.52	385.25
	North East and Yorkshire	16,230 (36.7)	367.44	9,445 (40.2)	401.66	25,675 (37.9)	379.33	374.69	383.97
	North West	6,580 (33.2)	332.16	31,265 (43.6)	436.42	37,845 (41.4)	413.83	409.66	418.00
	South East	5,390 (32.7)	327.06	22,080 (36.7)	367.05	27,470 (35.8)	358.45	354.21	362.69
	South West	10,050 (33.5)	334.89	7,450 (34.6)	346.43	17,500 (34)	339.71	334.67	344.74
	Unknown	90 (32.7)	327.27	0 (NaN)	NA	90 (32.7)	327.27	259.66	394.89

Figures

Figure S1: Monthly rate of **dementia patients** issued an antipsychotic in TPP (blue line) and EMIS (red line) practices; (a) shows the rates of all antipsychotic while (b) separates out the antipsychotic medications into four common types. Grey lines represent the start of the coronavirus lockdowns in England.

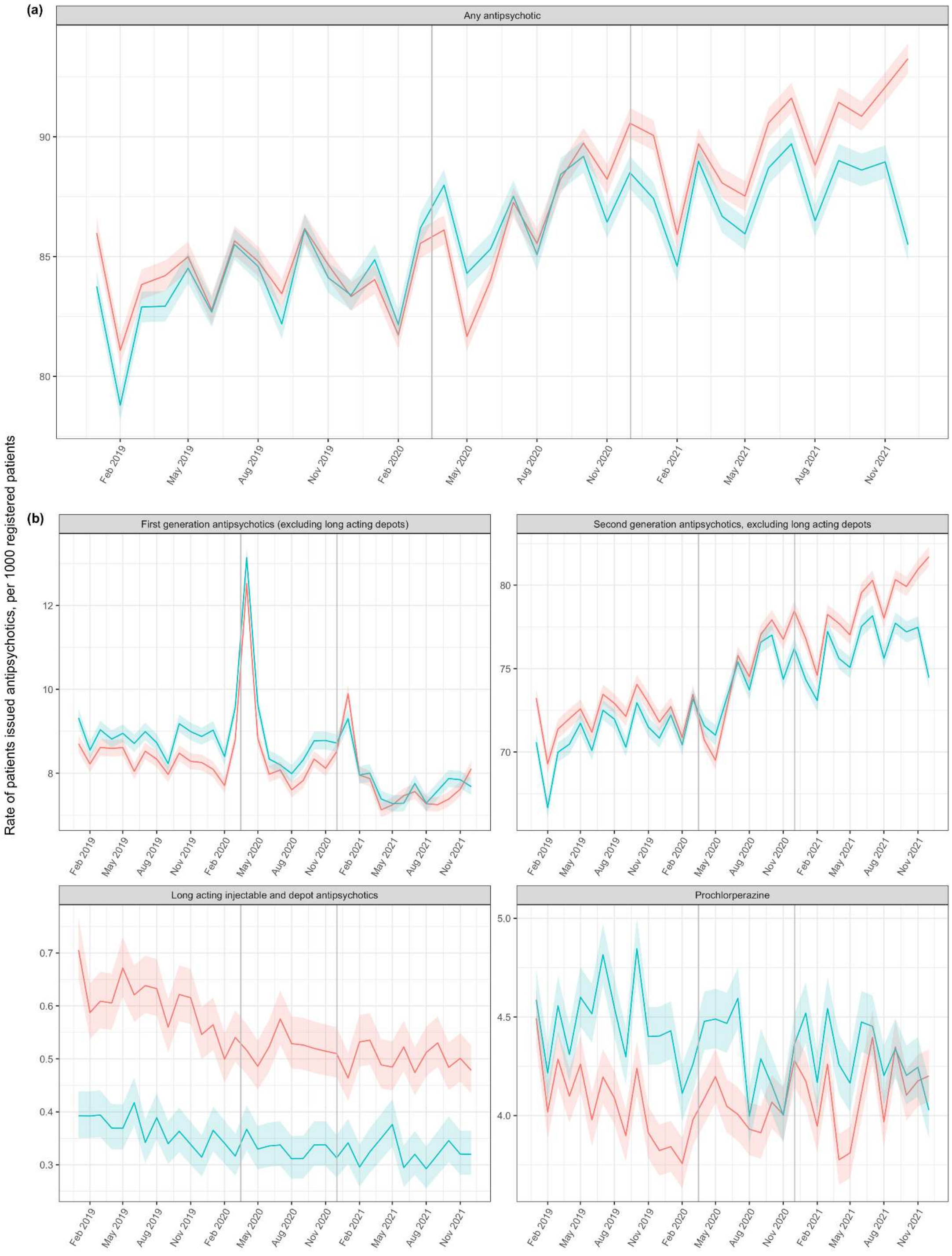

Figure S2: Monthly rate of **dementia patients newly issued** an antipsychotic in TPP (blue line) and EMIS (red line) practices; (a) shows the rates of all antipsychotic while (b) separates out the antipsychotic medications into four common types. Grey lines represent the start of the coronavirus lockdowns in England.

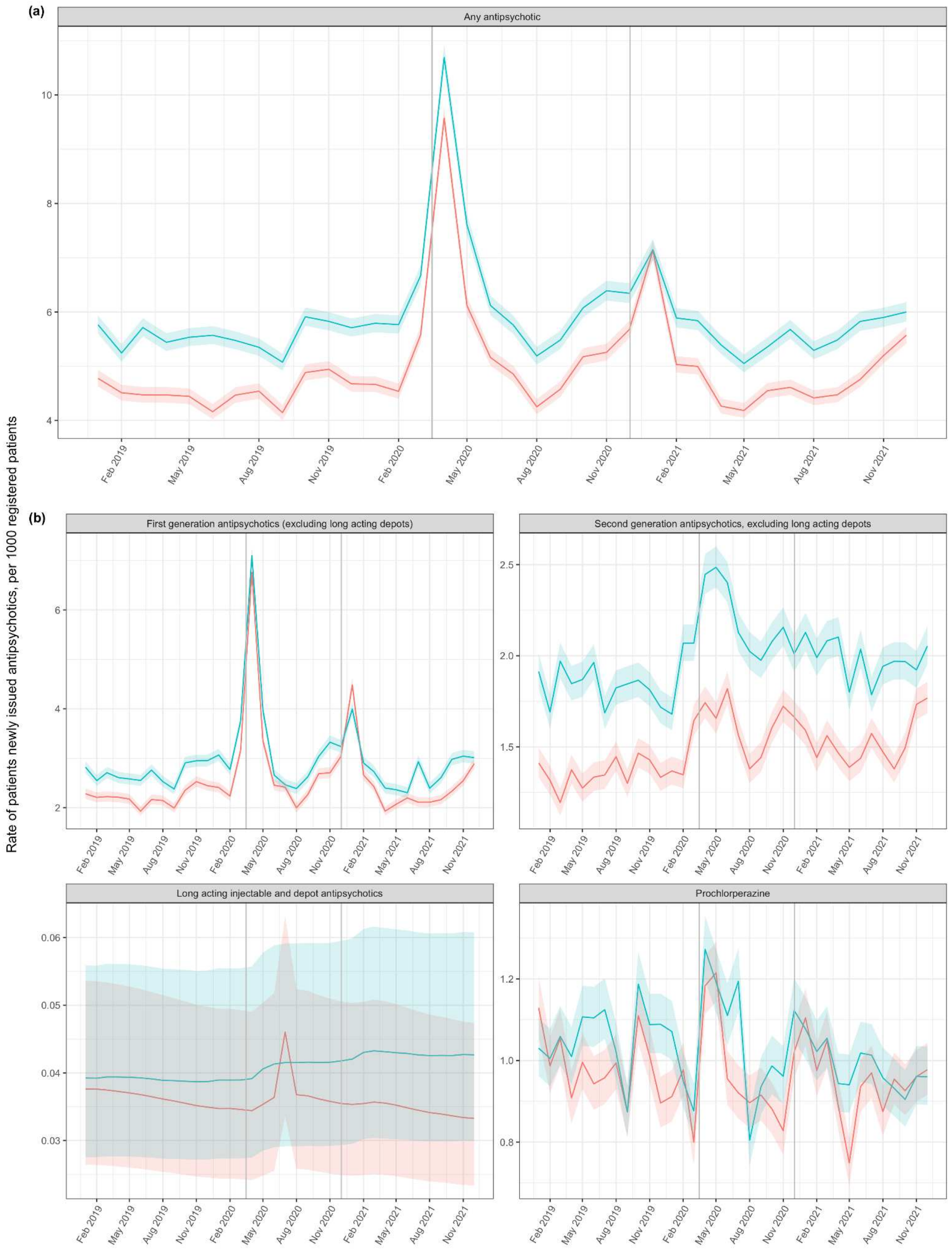

Figure S3: Monthly rate of **care home patients** issued an antipsychotic in TPP (blue line) and EMIS (red line) practices; (a) shows the rates of all antipsychotic while (b) separates out the antipsychotic medications into four common types. Grey lines represent the start of the coronavirus lockdowns in England.

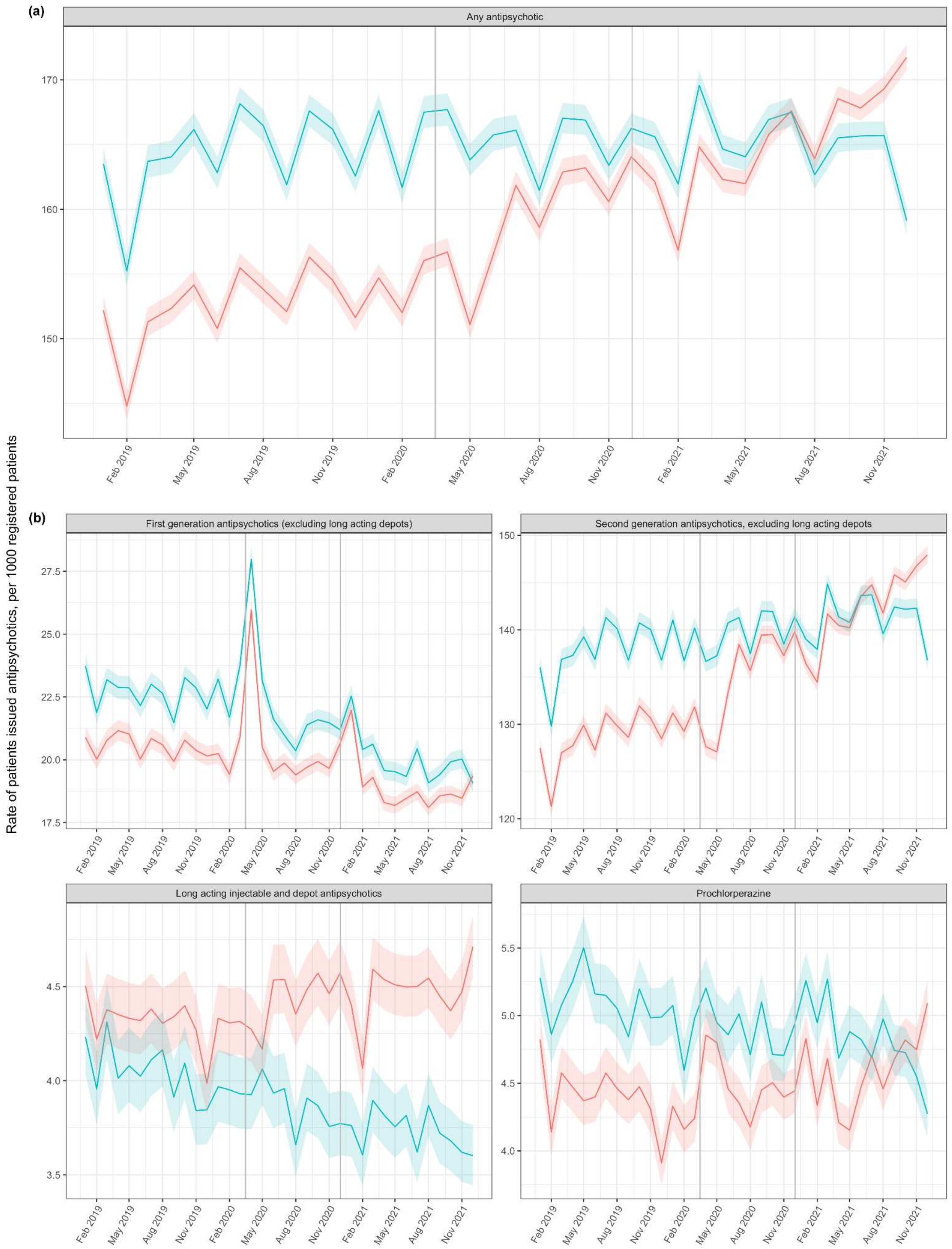

Figure S4: Monthly rate of **care home patients newly issued** an antipsychotic in TPP (blue line) and EMIS (red line) practices; (a) shows the rates of all antipsychotic while (b) separates out the antipsychotic medications into four common types. Grey lines represent the start of the coronavirus lockdowns in England.

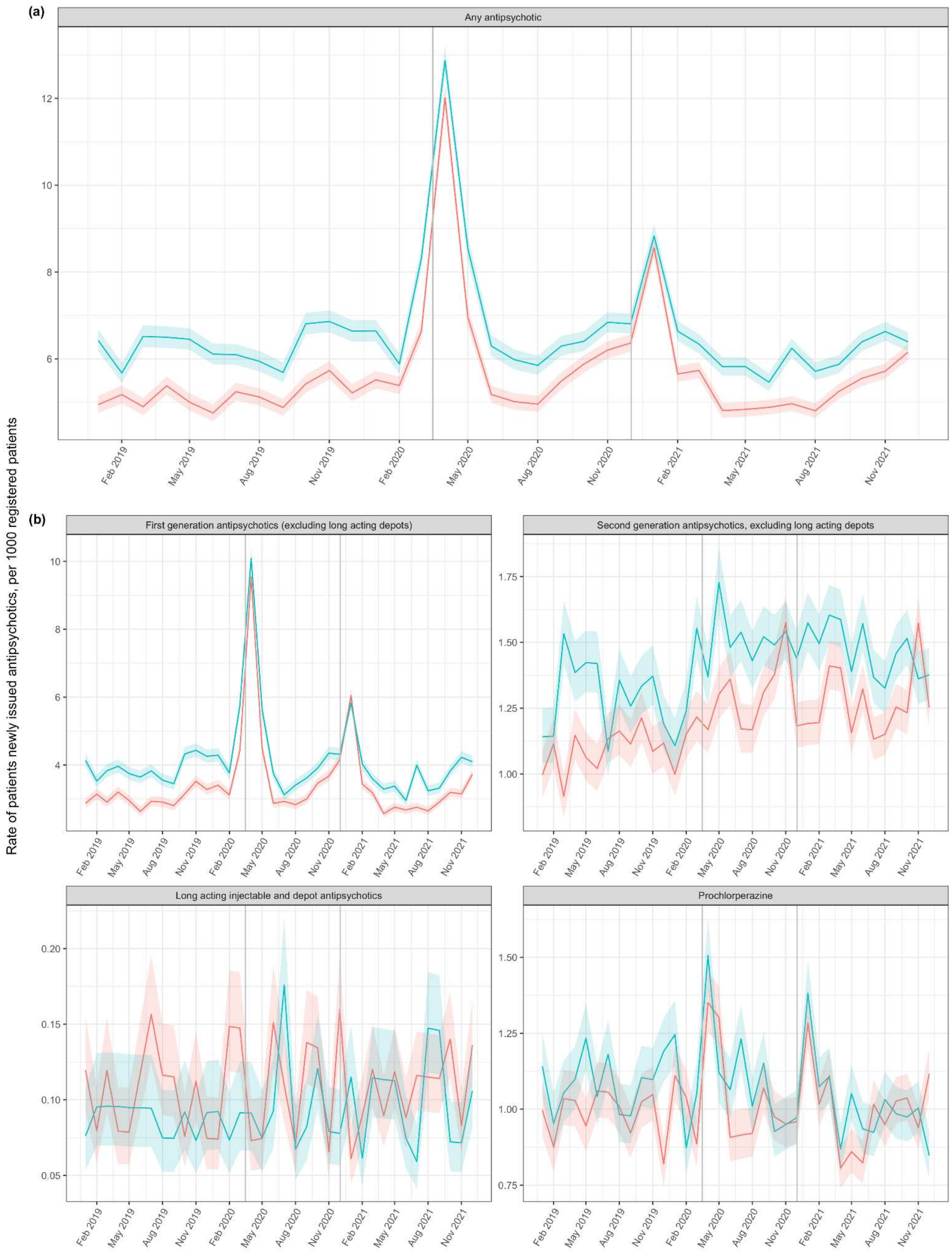

Figure S5: Monthly rate of **patients with a learning disability** issued an antipsychotic in TPP (blue line) and EMIS (red line) practices; (a) shows the rates of all antipsychotic while (b) separates out the antipsychotic medications into four common types. Grey lines represent the start of the coronavirus lockdowns in England.

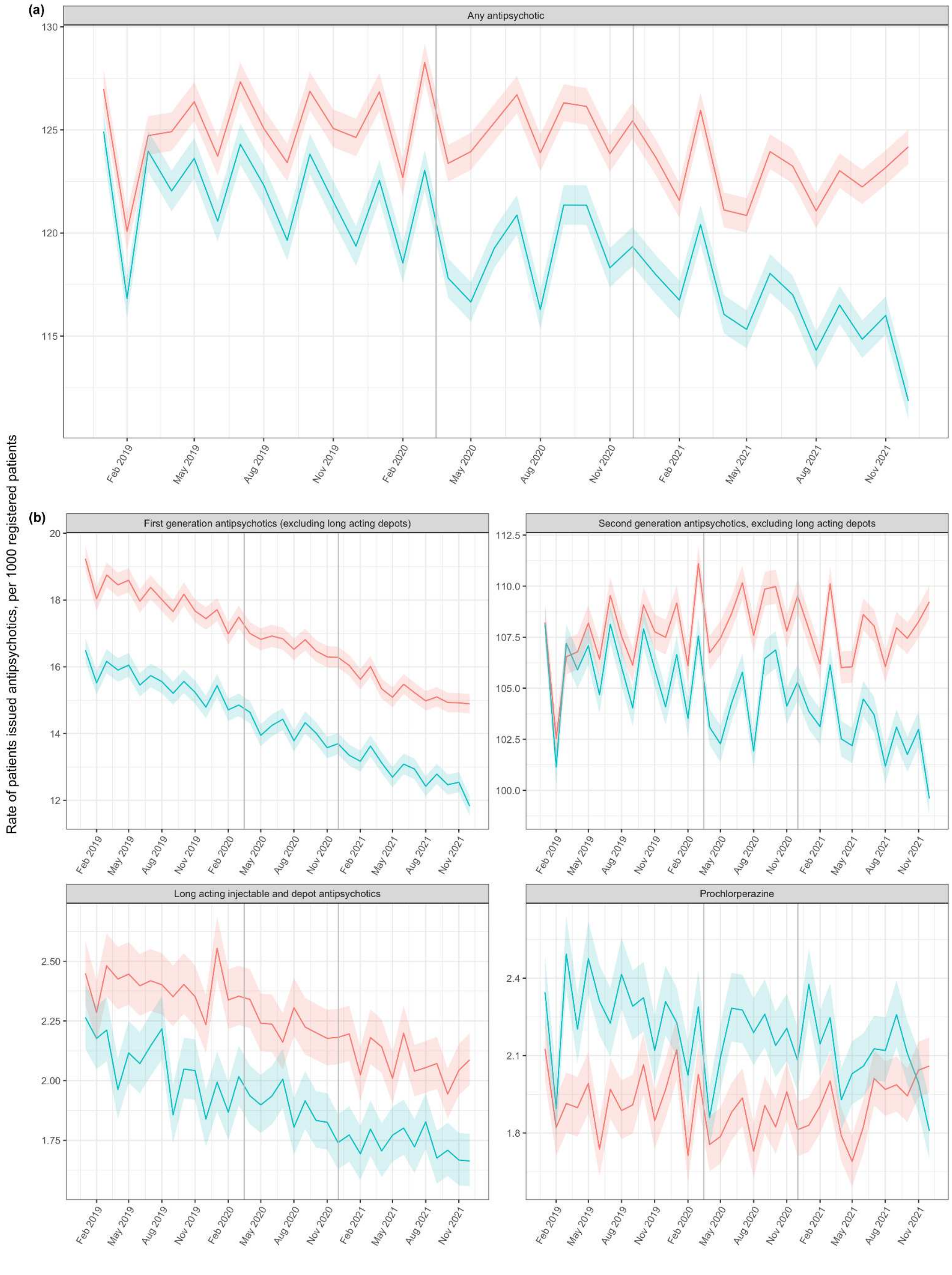

Figure S6: Monthly rate of **patients with a learning disability newly issued** an antipsychotic in TPP (blue line) and EMIS (red line) practices; (a) shows the rates of all antipsychotic while (b) separates out the antipsychotic medications into four common types. Grey lines represent the start of the coronavirus lockdowns in England.

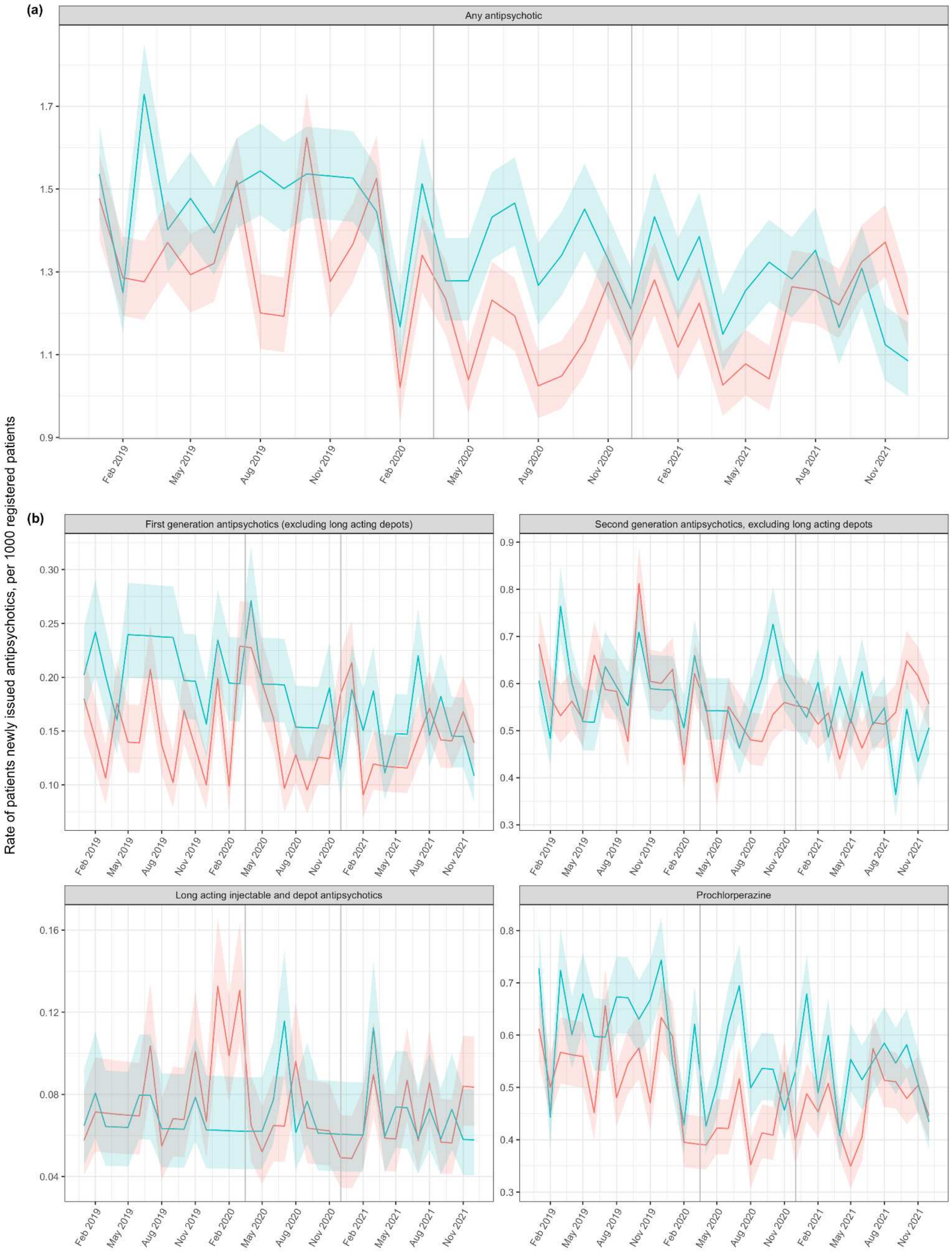

Figure S7: Monthly rate of **patients with autism** issued an antipsychotic in TPP (blue line) and EMIS (red line) practices; (a) shows the rates of all antipsychotic while (b) separates out the antipsychotic medications into four common types. Grey lines represent the start of the coronavirus lockdowns in England.

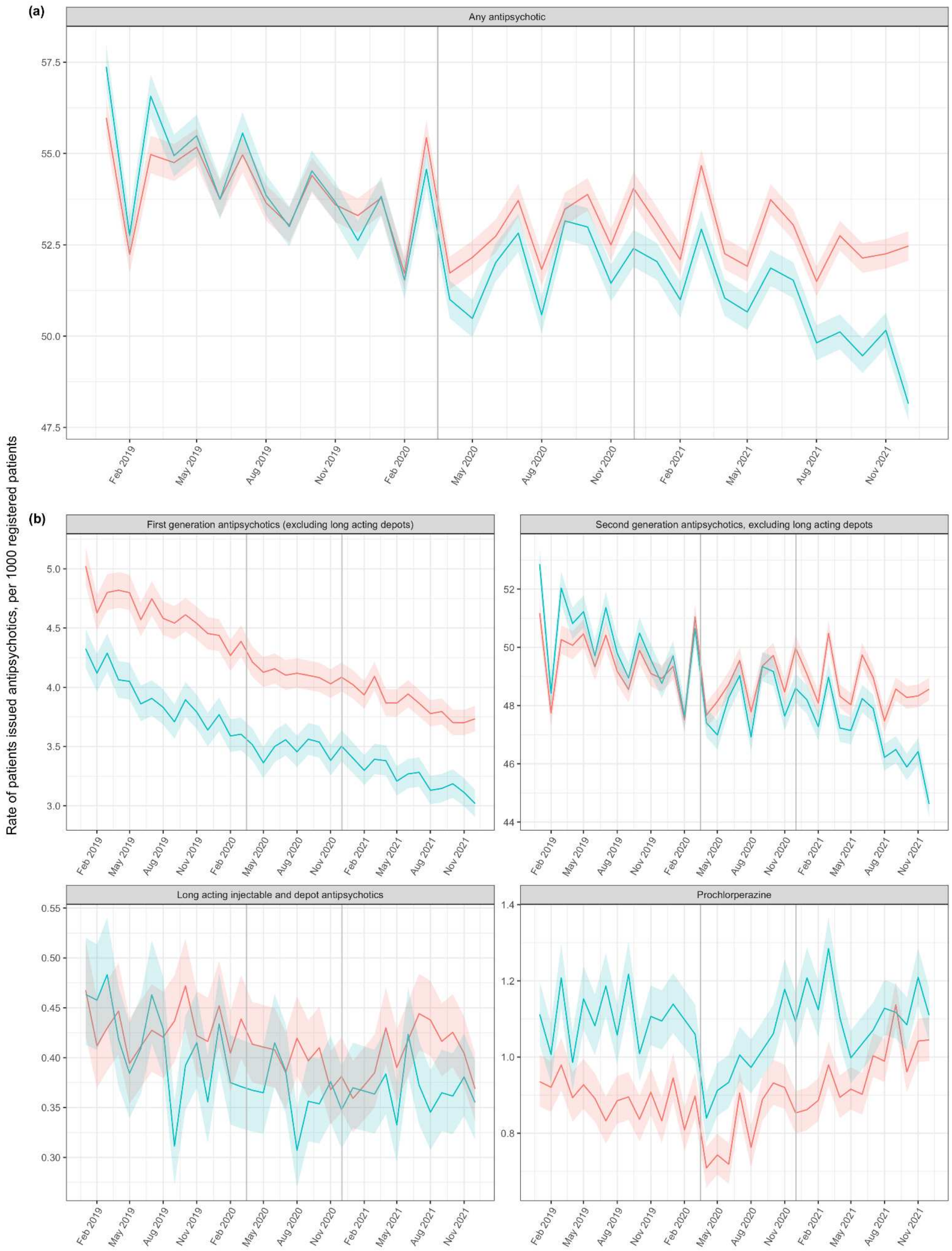

Figure S8: Monthly rate of **patients with autism newly issued** an antipsychotic in TPP (blue line) and EMIS (red line) practices; (a) shows the rates of all antipsychotic while (b) separates out the antipsychotic medications into four common types. Grey lines represent the start of the coronavirus lockdowns in England.

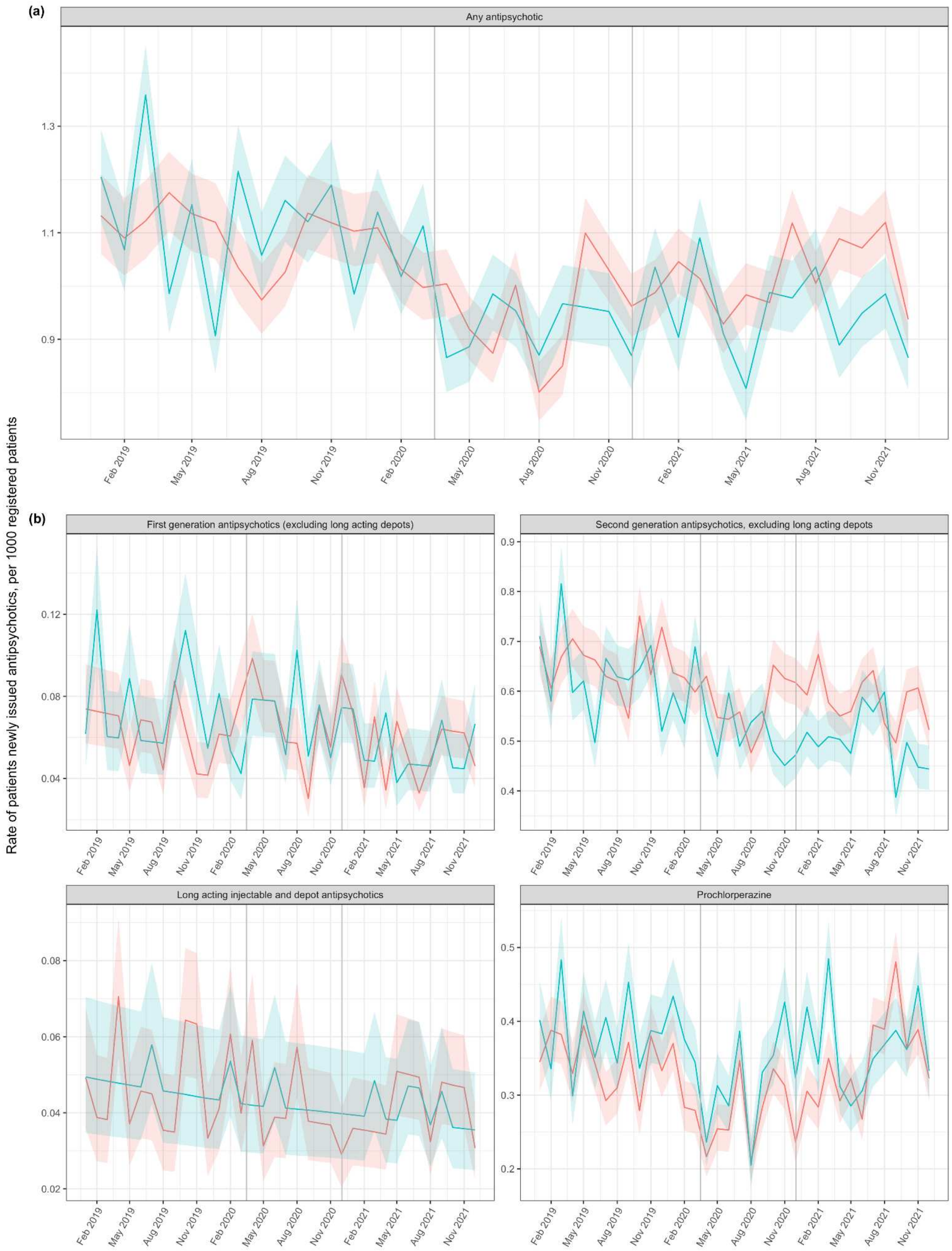

Figure S9: Monthly rate of **patients with a severe mental illness** issued an antipsychotic in TPP (blue line) and EMIS (red line) practices; (a) shows the rates of all antipsychotic while (b) separates out the antipsychotic medications into four common types. Grey lines represent the start of the coronavirus lockdowns in England.

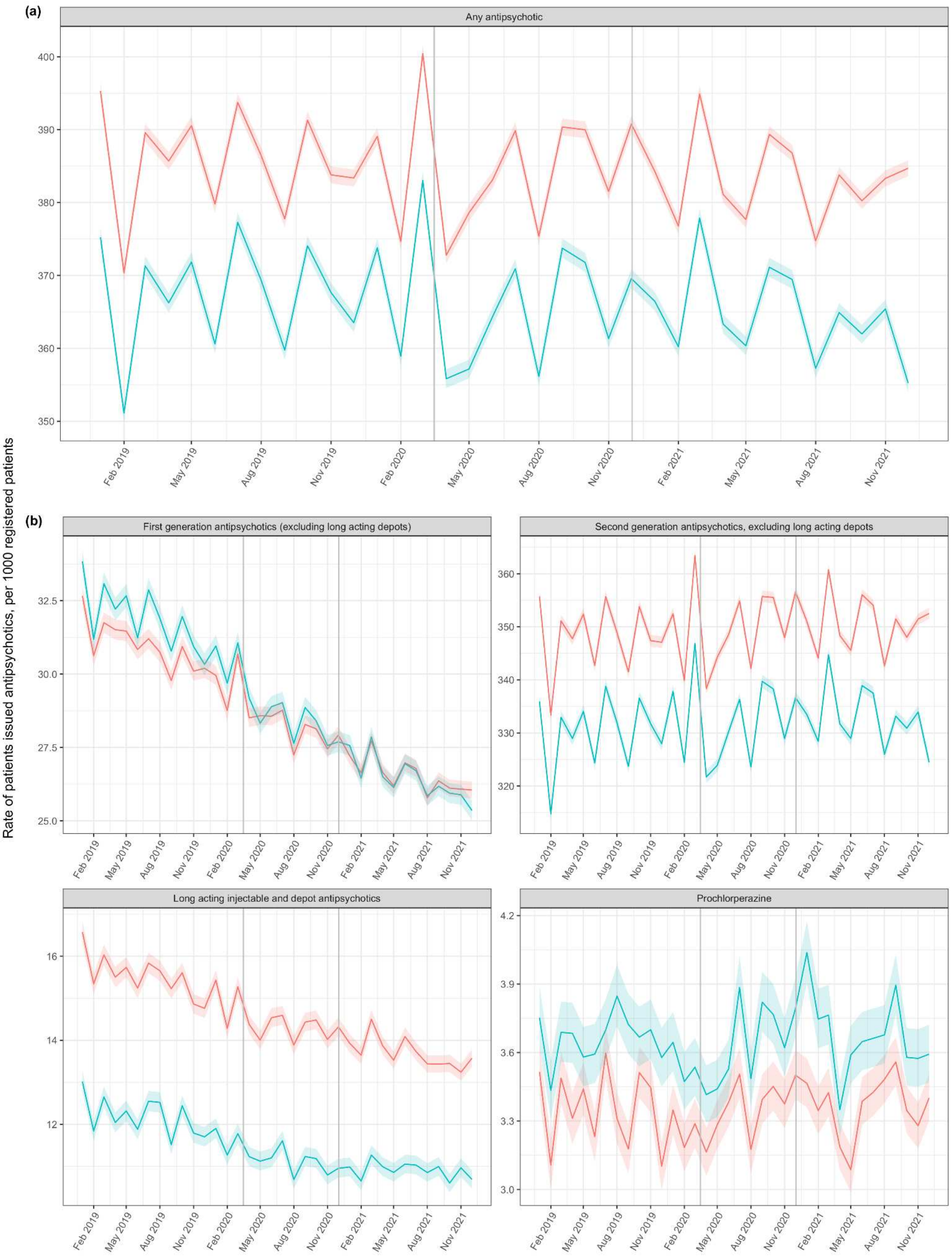

Figure S10: Monthly rate of **patients with a severe mental illness newly issued** an antipsychotic in TPP (blue line) and EMIS (red line) practices; (a) shows the rates of all antipsychotic while (b) separates out the antipsychotic medications into four common types. Grey lines represent the start of the coronavirus lockdowns in England.

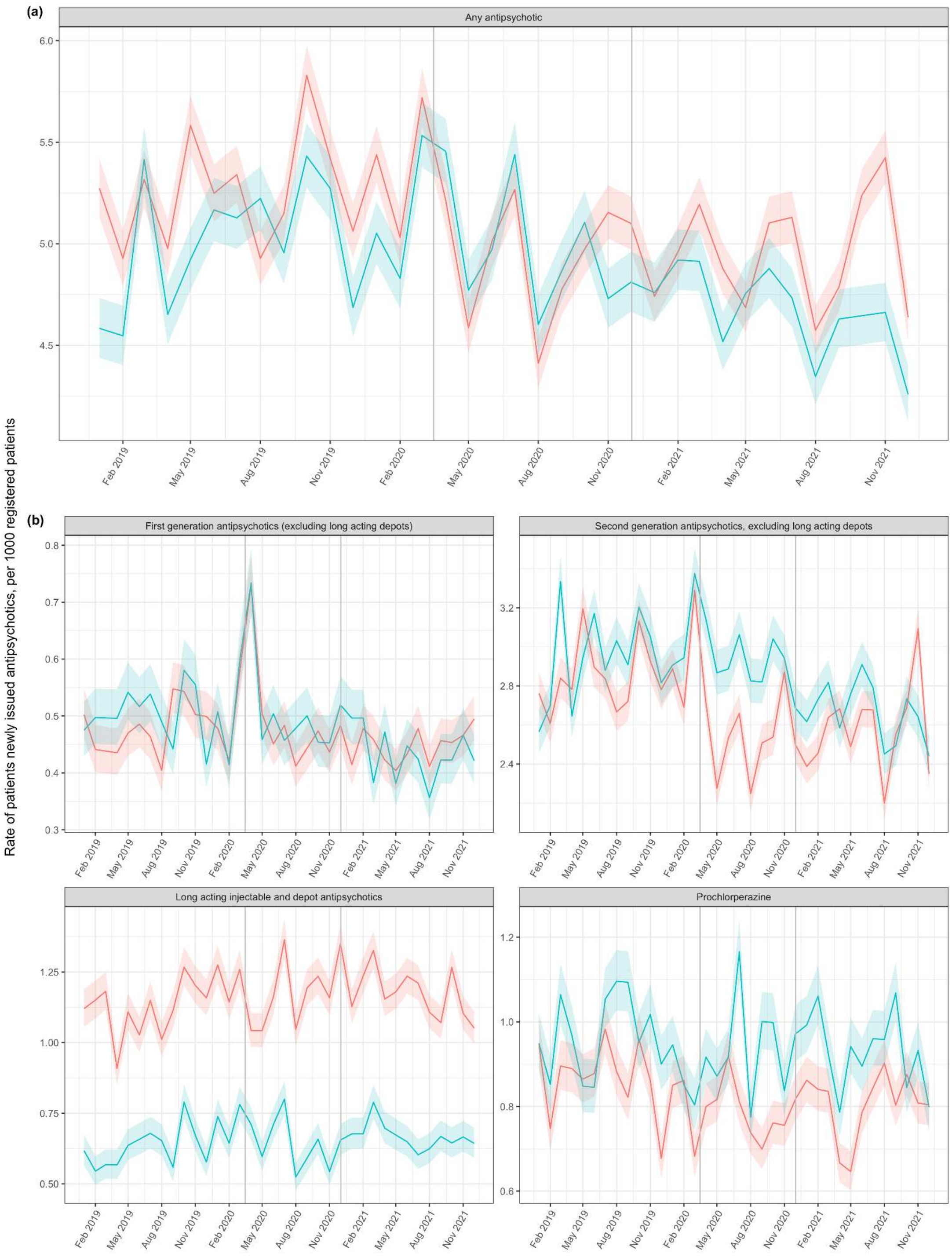

Supplement: Supplementary data [file bmjment-2023-300775supp001.pdf]
